# Machine Learning and Enhanced Sampling Simulations for Computing the Potential of Mean Force and Standard Binding Free Energy

**DOI:** 10.1021/acs.jctc.1c00177

**Published:** 2021-07-14

**Authors:** Martina Bertazzo, Dorothea Gobbo, Sergio Decherchi, Andrea Cavalli

**Affiliations:** † Computational & Chemical Biology, Fondazione Istituto Italiano di Tecnologia, via Morego 30, 16163 Genoa, Italy; ‡ Department of Pharmacy and Biotechnology (FaBiT), Alma Mater Studiorum − University of Bologna, via Belmeloro 6, 40126 Bologna, Italy; § BiKi Technologies s.r.l., Via XX Settembre 33/10, 16121 Genoa, Italy

## Abstract

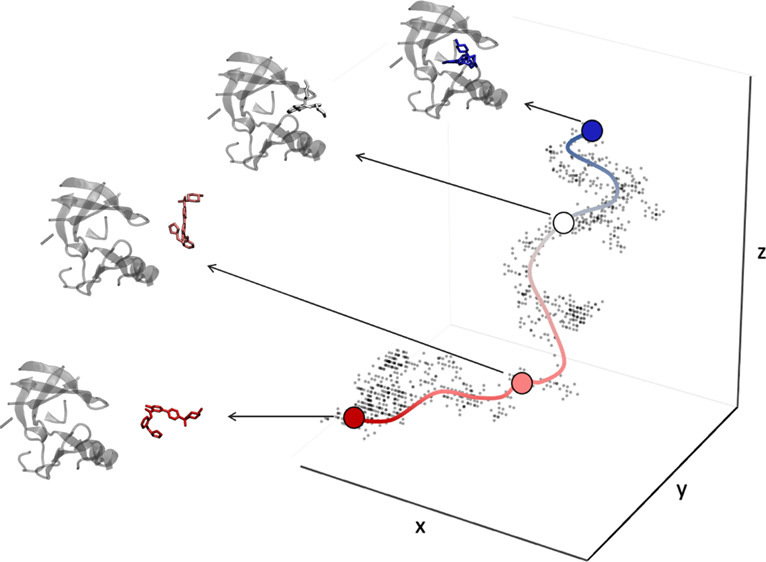

Computational capabilities are rapidly increasing, primarily because of the availability of GPU-based architectures. This creates unprecedented simulative possibilities for the systematic and robust computation of thermodynamic observables, including the free energy of a drug binding to a target. In contrast to calculations of relative binding free energy, which are nowadays widely exploited for drug discovery, we here push the boundary of computing the binding free energy and the potential of mean force. We introduce a novel protocol that leverages enhanced sampling, machine learning, and ad hoc algorithms to limit human intervention, computing time, and free parameters in free energy calculations. We first validate the method on a host–guest system, and then we apply the protocol to glycogen synthase kinase 3 beta, a protein kinase of pharmacological interest. Overall, we obtain a good correlation with experimental values in relative and absolute terms. While we focus on protein–ligand binding, the strategy is of broad applicability to any complex event that can be described with a path collective variable. We systematically discuss key details that influence the final result. The parameters and simulation settings are available at PLUMED-NEST to allow full reproducibility.

## 1 Introduction

In (bio)chemistry, free energy is still the most relevant and challenging physiochemical parameter to predict computationally. When studying the formation of biomolecular complexes under equilibrium conditions, the binding free energy is directly related to the affinity of the interacting partners. In drug discovery, accurate binding free energy estimations (within 1 kcal/mol) are crucial to identifying novel drug candidates.^1,2^ As such, a significant portion of the computer-aided drug discovery community is working to improve the accuracy, precision, and robustness of binding free energy predictions by refining the force field parameters^3−9^ and enhancing the sampling of slow degrees of freedom.^10,11^ In this context, enhanced sampling algorithms^12,13^ are increasingly combined with machine learning for more accurate free energy predictions.^14−17^

The advances in free energy perturbation (FEP) have enabled the frequent application of FEP in drug discovery to estimate the relative binding free energy (RBFE).^18−20^ When FEP simulations are applied to predict RBFEs, the ligand is alchemically transformed into another one through intermediate steps. Because free energy is a state function, the choice of the intermediate states is arbitrary, making the approach very flexible.^21,22^ Recent progress in computer hardware and software has made it feasible to apply FEP (or other alchemical) methodologies to absolute binding free energy (ABFE) predictions,^1,23−26^ creating the possibility of directly comparing the binding affinities across chemically different molecules that bind the same target or targets of the same family.^27,28^ Although attractive, the routine application of FEP approaches to ABFE calculations is still limited because they do not fully consider how key phenomena (e.g., induced fit and desolvation) contribute to the binding affinity.^29,30^ Their broad application to drug discovery is also limited by the higher computational cost of ABFE relative to RBFE studies. Additionally, FEP provides minimal details about binding intermediates, transient pockets, and molecular mechanisms because these calculations rely on unphysical paths (i.e., the alchemical transformations).

**Figure 1:**
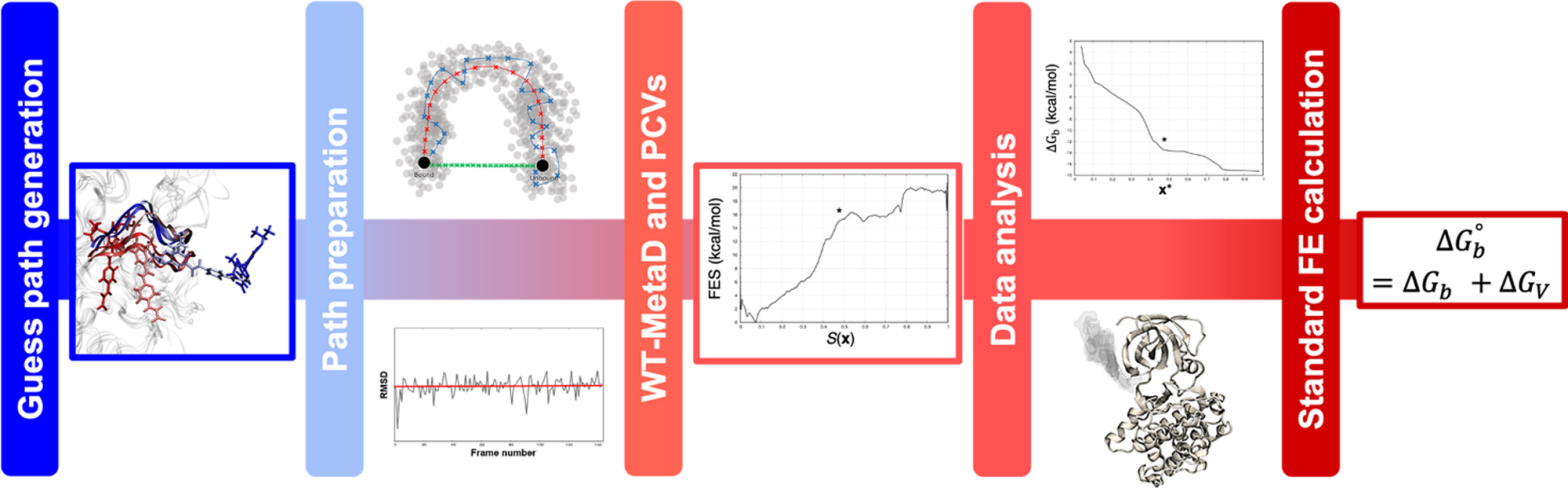
Graphical summary of the operational workflow.

A comprehensive representation of protein–ligand binding events can be provided by free energy methods based on physical paths, including steered molecular dynamics (MD) (via Jarzynski’s equation^31^), umbrella sampling,^32^ metadynamics,^33,34^ and so on. With these methods, one can simulate the complete association/dissociation process of a drug binding to a target in explicit water, taking the structural flexibility of the receptor into account. The free energy is then calculated from the potential of mean force (PMF), leading to good thermodynamic and kinetic estimations.^35^ Although attractive, there are at least two key challenges to be addressed to make these approaches widely applicable to drug discovery: (i) the choice of the “optimal” collective variables (CVs) to recapitulate the physical path and capture the slow degrees of freedom of the system and (ii) the definition of the operational workflow to set up, run, and analyze the simulations and eventually provide thermodynamics and kinetics data. For point (i), we here exploit the path CVs (PCVs),^36^ which have been extensively used to study protein–ligand binding. For point (ii), a simple, complete, and semiautomatic approach to path-based applications has not yet been reported, although attempts in this direction date back to 2010.^37^ We here devise an operational workflow that encompasses: (i) an enhanced sampling method to generate an initial guess path (we use adiabatic bias molecular dynamics unbinding simulations (ABMD)^38^ or steered MD); (ii) a machine-learning method to extract an approximate minimum free energy path from the initial guess path; (iii) a steered MD-based ad hoc method here introduced to make uniform the root mean square displacement (RMSD) between consecutive frames to eventually define the PCVs; (iv) well-tempered metadynamics^39^ (WT-MetaD) using the PCVs to obtain the PMF; (v) a technique based on the solvent excluded surface to compute the standard volume correction via NanoShaper;^40^ and (vi) the calculation of the standard binding free energy via the ratio of partition functions. We discuss this last aspect as reported by Doudou et al.,^41^ because it requires identifying the frame discriminating between the bound and unbound states, and this choice influences the outcome. Here too, we suggest a system-independent and semiautomatic procedure to identify the most reliable separating frame by analyzing the binding free energy profile. The pipeline depicted in Figure 1 is validated by computing the binding affinities of two diverse series of compounds, targeting two well-known benchmark systems, namely, a host–guest (HST–GST) complex^43^ and the glycogen synthase kinase 3 beta (GSK-3β),^44^ a system of pharmacological interest. The computational results and experiments correlated well. In addition to evaluating the RBFE correlations, we also discuss the accuracy of our estimates in absolute terms. This semiautomatic method is of wide applicability for path-based free energy methods, limiting the number of free parameters and human intervention.

## 2 Methods

Here, we introduce a computational strategy to compute the PMF along the PCVs.^36^ While the proposed protocol is of general applicability, we focus on the protein–ligand binding free energy. The procedure is detailed in the following paragraphs, and it can be summarized in the main steps listed below:

i) Generation of a MD trajectory describing the rare event under investigation, for example, the association/dissociation of protein–ligand complexes;
ii) Identification of a preliminary minimum free energy path by a machine-learning path-finding algorithm^42^ and optimization of the distance between consecutive frames (i.e., RMSD) by the equidistant waypoints algorithm (reported here for the first time);
iii) Reconstruction of the PMF by WT-MetaD on PCVs;
iv) Estimation of the standard binding free energy by processing the PMF plus the standard volume correction via a NanoShaper-based^40^ technique, purposely developed for the present study.

### 2.1 Path Generation via Enhanced MD Simulations

The characterization of the free energy profiles underlying binary complexes’ dissociation processes was considered a testbed for our computational strategy. In particular, we considered two sets that have been well characterized by both experiments and computations: the cucurbit[8]uril (CB8) HST–GST system proposed in the SAMPL6 challenge and a congeneric series of ATP-competitive inhibitors against the GSK-3β.^43,44^ In Section 2.5, we report the standard protocol used to set up both systems. To generate the putative dissociation paths connecting the bound and unbound states of the ligand, steered MD^45,46^ and ABMD^38^ simulations were performed on the CB8 and GSK-3β systems, respectively. In the steered MD simulations of the HST–GST complexes, the center of mass (COM) of the guest (GST) was pulled out from the CB8 cavity by applying a spring constant of 2000 kJ mol^–1^ nm^–2^ and a pull rate of 0.0001 nm ps^–1^, except for G2 and G3, whose larger molecular structures required a slightly higher pull rate. When asymmetric GST molecules are involved, there are slight differences between the two PMF profiles projected over both exit directions from the CB*n* cavity, as reported in previous studies on CB*n* complexes.^47,48^ Hence, we performed two independent steered MD simulations along the two exit directions from CB8 for the asymmetric GST molecules included in our selection (G2 and G3). After 10 ns of steered MD simulations, a final COM distance between CB8 and the GST molecules of approximately 10 Å was achieved for all complexes. GROMACS 2016.5^49^ was used to perform the pulling simulations in the NVT ensemble. The dissociation processes for the congeneric series of pyrazine-derivative inhibitors of GSK-3β were previously studied by ABMD coupled with an electrostatics-driven CV (i.e., elABMD).^50^ elABMD is an enhanced sampling simulation technique that smoothly drives the system toward the desired end state while minimally perturbing its natural evolution, determined by thermal fluctuations because of finite temperature.^38^ As such, once the system-dependent force constant affecting the magnitude of the backward fluctuations of the reaction coordinate is tuned correctly, a rare event can be accurately sampled, including the metastable states. Here, we selected one of the 20 unbinding trajectories reported in ref (50). by considering: (i) the computational unbinding time near the average one, as defined in ref (49).; (ii) the achievement of the complete ligand solvation (assessed in this study by looking at the protein–ligand contacts within 6 Å); and (iii) the physical soundness of the dissociation pathway. Here, we evaluated the exit direction of the ligand and the time spent sampling the unbound, prebound, and bound states, whose relevance to this study is discussed in Section 4.

### 2.2 Approximate Minimum Free Energy Path Finding and Optimization of the Interframe Distance with the Equidistant Waypoints Algorithm

At this stage, enhanced sampling (or plain MD) trajectories are already available. To accelerate the path-building phase, we do not refine the path by running further simulations. Instead, we clean up the path using the available samples; that is, we find an approximate minimum free energy path. This strategy is very flexible because it does not require running further simulations (e.g., the string method), and it can deal with presampled trajectories from plain MD or enhanced sampling (in the latter case, more care is needed). The execution time of this step is negligible compared to a MD run. To find an approximate minimum free energy path, we use the principal path algorithm, as previously formulated.^42^ Given a points cloud, this machine-learning method connects two points defined a priori in data space and tries to pass through the local support of the data distribution, capturing the most “abstract” morphing path between these points. The method searches for a smooth out-of-sample geodetic ruled by the data sample density. It was inspired by the string method,^51^ but with several differences:

- The string method is an online method similar to a stochastic gradient descent which is run simultaneously to a MD simulation. The principal path method is a batch method and is applied a posteriori irrespective of the MD sampling technique.
- The principal path can be applied to any kind of data, provided a points cloud or distance matrix is available, making it a machine-learning method, particularly a kernel method.
- The string method has no variational formulation, whereas a functional form is explicit in the principal path method. Indeed, we have shown that the string method iterations minimize the principal path functional in an approximate way.^42^

Finally, the method might also be seen as a plain elastic band,^52^ where the potential function is substituted with the k-means cost function (discussed below).

The principal path algorithm is formally a regularized version of the k-means clustering algorithm. However, its purpose is significantly different as the principal path searches for a smooth one-dimensional manifold discretized by the waypoints. In particular, if we consider a set of points *X* = {*x_j_* ∈ *R^d^*}, *j* = 1, .... , *N*, and two points *w*_0_, *w*_*N_c_* + 1_ ∈ *R^d^*,the path connecting these two points is defined as an ordered set *W* of *N_c_* waypoints *w* ∈ *R^d^*. The principal path is found by minimizing the standard k-means cost function with the addition of a quadratic regularization term, which restrains the distance between consecutive waypoints and controls the level of smoothness of the path:
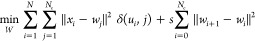
1where δ(*u_i_*, *j*) is the Kronecker delta, which gives the membership of the *i*th sample to *j*th cluster/waypoint and *u_i_* is a membership function that gives the cluster index; hence δ(*u_i_*, *j*) is different from 0 only if the *i*th sample belongs to the *j*th cluster. The first term coincides with the standard *k*-means cost function, and the second term introduces a set of harmonic restraints applied to consecutive waypoints. The hyper-parameter *s* regulates the trade-off between the data fitting and the smoothness of the path, as shown in Figure S1 in the Supporting Information.

**Figure 2:**
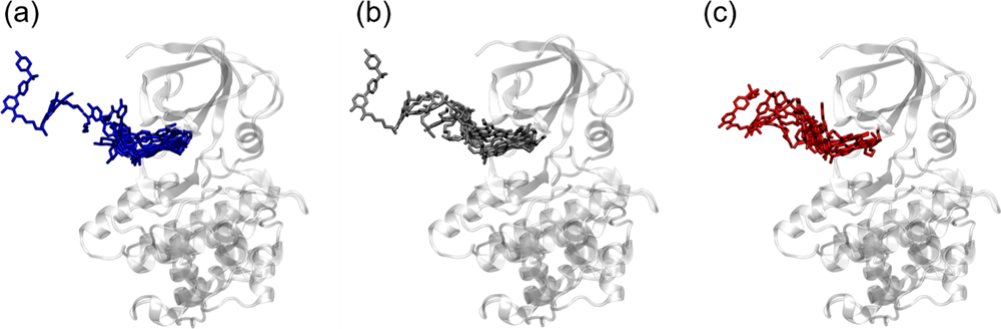
Graphical representation of the path preparation for one of the GSK-3β complexes studied in this work. (a) Guess unbinding path generated via elABMD; (b) approximate minimum free energy path defined via the path-finding algorithm; (c) optimized unbinding path in terms of spacing between pairs of successive frames. A reduced number of frames are reported to simplify the representation of the system in the three stages.

By applying the path algorithm to real conformations sampled by MD, the closest physical frames of the original simulation to the ones calculated by the principal path are identified, thus defining a complete sequence of consecutive conformations capturing the event sampled by MD. At this stage, a clean path is available, but because of the peculiarities of molecular simulations, the distance in terms of RMSD between the neighboring snapshots identified is far from being uniform (Figure 2b). This aspect appears to be particularly relevant when the PCVs,^36^*S*(***x***) and *Z*(***x***), are chosen to trace the principal path. As reported in the original paper introducing the PCVs,^36^ consecutive frames must be as equidistant as possible to ensure the smooth progression of *S*(***x***) along the path and, more importantly, the proper mapping between the formal variable *S*(***x***) and the underlying metric space. Thus, we devised an algorithm based on a series of 20 ps-long steered MD simulations to make uniform the spacing between pairs of successive frames by placing additional and equidistant configurations as needed (Figure 2c). This automated procedure is close in spirit, even if devised independently, to a procedure reported by Bernetti et al.^53^ The principal path algorithm was developed in MATLAB, and the algorithm to make uniform the distance between consecutive frames was developed in Python 3 (see Algorithm 1 pseudocode in the Supporting Information). The code is available upon request. The steered MD simulations were run in GROMACS 2016.5^49^ patched with PLUMED 2.5.^54^

The interframe distance was computed as the RMSD of the heavy atoms of the GST molecule for the CB8 complexes, while the RMSD alignment was performed on a selection of 16 C atoms defining the ring core of the CB8 molecule. For the GSK-3β system, the heavy atoms of the ligand and the protein residues located within 4 Å of the ligand in the bound state were considered for RMSD computation. For alignment, we used 25 C_α_ atoms belonging to residues that were uniformly distributed on the protein structure showing a small coordinate displacement during a plain MD simulation. The GSK-3β residues considered in this study for the RMSD alignment were Met162, Tyr163, Gln164, Leu165, Phe166, Arg167, Ser168, Leu169, Ala170, Tyr171, Ile172, Ser237, Ile238, Asp239, Val240, Trp241, Ser242, Ala243, Gly244, Cys245, Leu320, Leu329, Pro331, Leu332, and Ala334 according to PDB code 4ACC. The target RMSD threshold between consecutive frames along the path was set equal to 1 Å for all systems.

### 2.3 WT-MetaD and PCVs

The free energy surfaces underlying the unbinding processes under investigation were reconstructed by WT-MetaD^39^ along the PCVs, *S*(***x***) and *Z*(***x***):
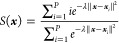
2and
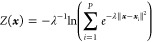
3

In eqs 2 and 3, ***x*** represents the current system configuration, ∥***x*** – ***x****_i_*∥^2^ is the distance between the current configuration and the *i*^th^ frame of the path, *P* is the number of frames included in the original path, and λ is a parameter that modulates the smoothness of the path representation. Here, λ was set equal to 230.0 nm^–2^ according to the following heuristic equation that only depends on quantities available before running WT-MetaD:
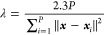
4

WT-MetaD was initialized from the bound state and then ran to a maximum of 1 μs. By fixing the maximum computing time to be invested in sampling the potential energy surfaces via WT-MetaD, we accumulated a total simulation time of 8 μs and ∼8.5 μs for the CB8 and GSK-3β systems, respectively. Gaussians with a nominal height of 0.2 kcal/mol were used together with a bias factor of 15. The width of the Gaussians was set to 0.2 and 0.01 nm^2^ along *S*(***x***) and *Z*(***x***), respectively, whereas the available space along the *Z*(***x***) dimension was limited by placing a wall at *Z*(***x***) equal to 0.05 nm^2^. The Gaussians deposition time was set to 500 MD steps. All WT-MetaD simulations were performed using GROMACS 2016.5^49^ patched with PLUMED 2.5.^54^ WT-MetaD simulations run on one GPU node (2 CPU Intel Xeon E5–2650 v4 @ 2.20GHz 12 Cores each, 2 NVIDIA Tesla P100-PCIE-12GB), performing 100 and 30 ns/day for the CB8 and GSK-3β systems, respectively. Simulation of one ligand of GSK-3β for 1 μs of WT-MetaD costs approximately 30 days of one node computing time.

### 2.4 Standard Binding Free Energy Computation

The standard binding free energy, Δ*G*_b_^°^, was calculated as reported by Doudou et al.:^41^
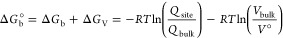
5

The first term of eq 5 represents the probability ratio between the bound and unbound states of the ligand. The second term is the standard volume correction. *Q*_site_ and *Q*_bulk_ denote the partition functions for the bound and unbound regions, respectively, whose ratio is computed by integrating the FES as reconstructed by WT-MetaD, F(*S*, *Z*), and defined to be zero at its lowest point (i.e., the ligand bound state) (eq 6). The frame separating the bound and unbound states was identified as the first inflection point obtained by plotting the binding free energy, Δ*G*_b_, as a function of the bound/unbound frame. In Sections 3.2.1 and 4, the procedure is further explained and discussed together with a representative example.

In detail, the argument of the logarithm in the first term is:
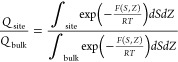
6

The second term of eq 5 quantifies the free energy for changing from the standard-state volume *V*° equal to 1661 Å^3^, corresponding to a concentration of 1 M, to the sampled unbound volume, *V*_bulk_. This correction term is needed to include in the free energy estimate the effect of the limited conformational space available to the ligand when in the unbound state. In this study, the unbound volume, *V*_bulk_, was quantified using NanoShaper^40^ (freely available at https://gitlab.iit.it/SDecherchi/nanoshaper and also available in the BiKi Life Sciences software package^55^), by considering the *S*(***x***) frames of the principal path describing the dissociated state of the binary complex. In detail, we collected all the frames belonging to the unbound state in a single pdb file, and then we computed the solvent excluded surface on this union of the ligand atoms. This union surface gives an accurate approximation of the volume traced by the ligand in the unbound state (Supporting Information). In the Results section, the standard binding free energy, Δ*G*^°^_b_, for each complex refers to the time average of the last portion of each WT-MetaD simulation, whose length was determined by two conditions:^56^ (i) in the considered window, the system is fully diffusive along *S*(***x***) and (ii) the residual height of the hills must be less than 10% of the initial height (i.e., 0.2 kcal/mol in this study). An estimate of the sampling error is computed as the standard error of the time fluctuation of Δ*G*^°^_b_ over the converged portion of each WT-MetaD simulation. All the data and PLUMED input files to reproduce the results are available on PLUMED-NEST (www.plumed-nest.org), the public repository of the PLUMED consortium,^57^ as plumID:21.004.

### 2.5 System Setup

All the CB8 complex structures were obtained from the SAMPL6 repository.^43^ The host and GST molecules were modeled according to the general Amber force field (GAFF)^58^ version 1.8. The AM1-BCC^59,60^ point charges were used as supplied by the SAMPL6 organizers. Water is described by the TIP4P-Ew model.^61^ Sodium and chloride ions are added to neutralize the system and to maintain the corresponding experimental ionic strength at which the experimental binding affinity was measured, that is, 25 mM Na_3_PO_4_ buffer at pH 7.4.^43^ All complexes are minimized by 5000 steepest descent steps and then equilibrated. The equilibration protocol requires the thermalization of the system at 300 K in three steps using the Bussi–Parrinello thermostat^62^ for a total of 0.3 ns of dynamics. Subsequently, 1 ns of MD in the NPT ensemble is performed until the average pressure of the system is equilibrated to 1 atm according to the Parrinello–Rahman barostat.^63^ All MD simulations are performed using GROMACS 2016.5^49^ patched with PLUMED 2.5.^54^ Production runs are performed in the NVT ensemble, setting 2 fs as the time step. Velocities are randomly assigned before each production run. A cutoff of 12 Å was used for nonbonded interactions, while long-range electrostatic interactions were treated with the particle mesh Ewald^64^ scheme, using a grid spacing of 1.6 Å. A temperature of 300 K was controlled using the V-rescale thermostat,^62^ while bond lengths for chemical bonds involving hydrogens were restrained to their equilibrium values with the LINCS algorithm.^65^ For the detailed protocol used to set up the GSK-3β systems, we refer the reader to ref (50).

## 3 Results

First, we describe the computational strategy applied to the HST–GST system (the CB8-G6 complex). We detail the procedure implemented to identify the *S*(***x***) frame to compute the rate of partition functions between the bound and unbound regions (eq 6). In the second part of this section, we outline the results for the GSK-3β complex system.

### 3.1 HST–GST Benchmark System

**Figure 3:**
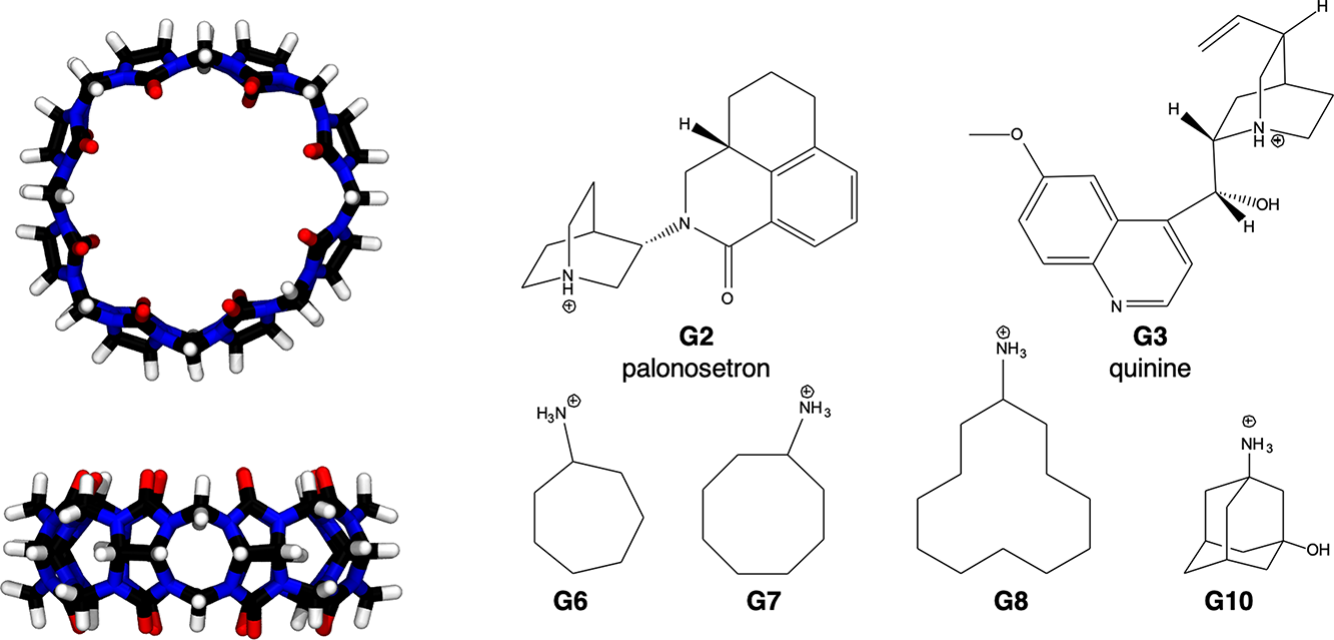
Top and side perspective views of the 3D structure of the CB8 host. Carbon atoms are represented in black, hydrogens in white, nitrogens in blue, and oxygens in red. GST molecules are shown as 2D chemical structures with an explicit protonation state.

As a testbed for our computational strategy, we chose the HST–GST system from the cucurbit[*n*]uril (CB*n*) family, proposed in the SAMPL6 binding challenge. We identified six positively charged GST molecules (Figure 3) displaying a wide range of binding affinities for the host, from −13.5 to −6.45 kcal/mol (Table 1), including a few cases whose experimental binding free energies differed by less than 1 kcal/mol. The highly symmetric CB8 comprises eight identical glycouril monomers linked by pairs of methylene bridges, resulting in its characteristic ring shape (Figure 3). Because of their top-bottom symmetry, asymmetric GSTs have at least two symmetry-equivalent binding modes. For this reason, G2 and G3 were included in our selection. All HST–GST complexes included in the data set display 1:1 experimental stoichiometry.

**Table 1:** Prioritization of the GST Molecules on Standard Binding Free Energies Obtained by WT-MetaD and ITC Experiments^a^

GST ID	Δ*G*_b_	Δ*G*_V_	Δ*G*^°^_b_	Rank_comp_	Δ*G*^°^_b,exp_	Rank_exp_
G2	–7.7	0.0	–7.7 ± 0.1	6	–7.66 ± 0.05	5
G3	–9.6	–0.1	–9.7 ± 0.1	3	–6.45 ± 0.06	6
G6	–9.3	0.4	–8.9 ± 0.1	5	–8.34 ± 0.05	3
G7	–10.8	0.3	–10.6 ± 0.1	2	–10.0 ± 0.1	2
G8	–12.9	0.4	–12.5 ± 0.1	1	–13.50 ± 0.04	1
G10	–9.4	0.2	–9.1 ± 0.1	4	–8.22 ± 0.07	4

a Free energy terms (i.e., Δ*G*_b,_ Δ*G*_V_, Δ*G*^°^_b_, and Δ*G*^°^_b,exp_) are reported in kcal/mol. Pearson correlation coefficient: 0.84. Bootstrap Pearson correlation coefficient: 0.72 ± 4e-5 (bootstrap standard error and 10,000 samples). Spearman coefficient: 0.6. RMSE: 1.5 kcal/mol. ME: 0.7 kcal/mol. The experimental data refer to Rizzi et al.^43^

### 3.2 HST–GST Binding Free Energy

As previously mentioned, steered MD was chosen as an enhanced simulation technique to generate preliminary paths of the HST–GST systems. The path algorithm^42^ was subsequently applied to identify an approximate minimum free energy path, describing the dissociation process for every complex in the benchmark data set, thus detecting the milestone frames. Once defined, the principal path was then subjected to the steered MD-based procedure to optimize the definition of the PCVs. Each optimized principal path was sampled for 1 μs with WT-MetaD combined with PCVs. Although the small size of the CB8 complexes could have required shorter simulations, we aimed to thoroughly inspect the statistics thanks to the relatively limited computational cost.

**Figure 4:**
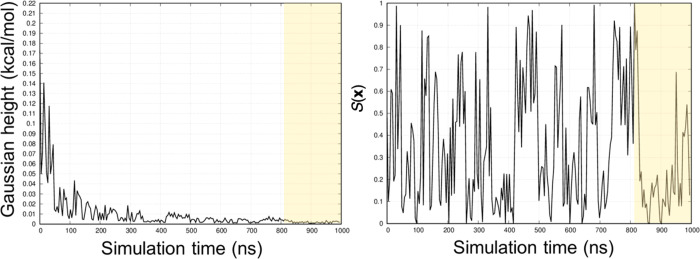
Gaussian hills height (left) and *S*(***x***) progression (right) as a function of the simulation time for the CB8-G6 complex. For consistency, the reaction coordinate was rescaled, setting *S*(***x***) = 0 (bound state) and *S*(***x***) = 1 (unbound state) for every complex reported in this study. The shaded region refers to the portion of the WT-MetaD simulation considered in the computation of the binding free energy.

The WT-MetaD convergence was primarily assessed by looking at the downward trend of the Gaussian hills height as a function of the simulation time and the diffusing behavior of the *S*(***x***) variable over the simulation time. For the former, a residual height of the hills of approximately 10% of the initial height was considered an acceptable threshold to assess the WT-MetaD convergence.^56^Figure 4 reports the Gaussian height and the progression of the *S*(***x***) variable as a function of the simulation time for the CB8-G6 complex. For this system, we established the convergence after 800 ns of simulation, when both conditions were fulfilled. In the Supporting Information, we also report the evolution of the Δ*G*_b_ along the simulation time, given its relevance when assessing the convergence of metadynamics-based simulations.

#### 3.2.1 Identification of the Bound/Unbound x* Frame

As outlined in eqs 5 and 6, the computation of the binding free energy requires the evaluation of the probability ratio between the bound and unbound states of the ligand, which in turn demands the identification of the *S*(***x***) frame discriminating the two states.

To establish a transferrable strategy for semiautomatically identifying the bound/unbound ***x*** frame (hereafter referred to as x*) for every CB8 complex in the data set, we monitored the behavior of the binding free energy, Δ*G*_b_, computed following eqs 5 and 6, changing the x* frame.

**Figure 5:**
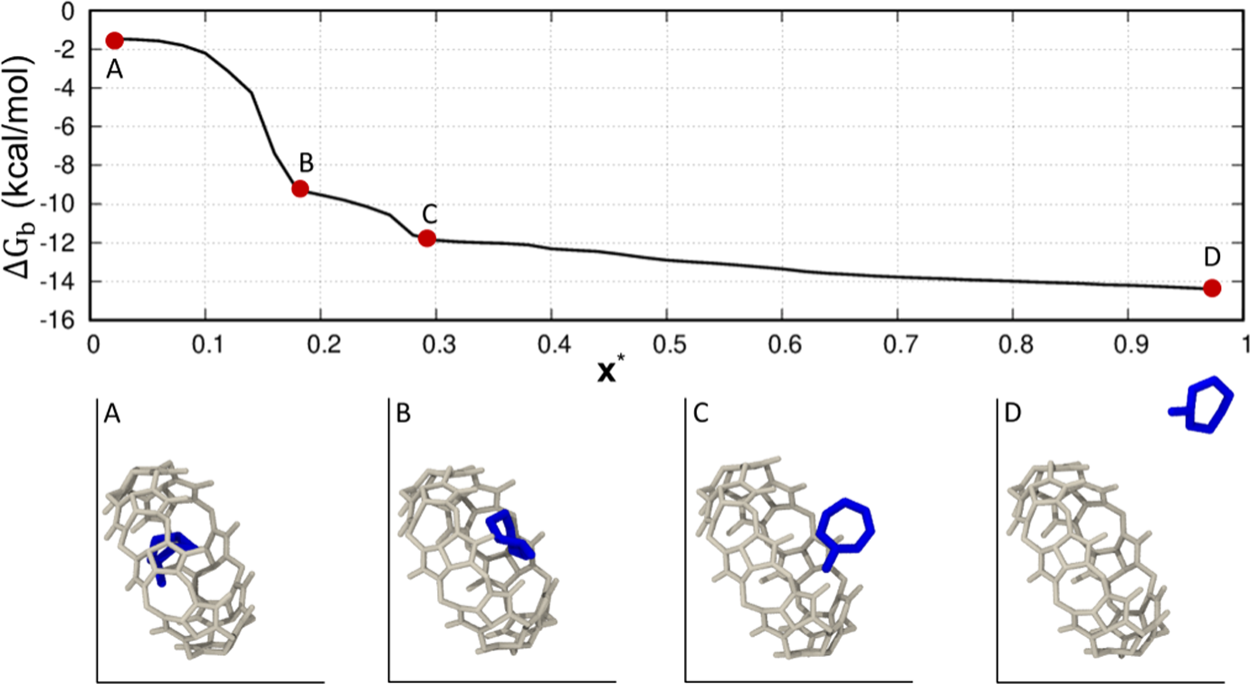
Sensitivity of the binding free energy, Δ*G*_b_, with varying x* frame for the CB8-G6 system.

Figure 5 reports the behavior of Δ*G*_b_ as a function of x* for the representative case of the CB8-G6 system, where the rescaled x* equal to 0 corresponds to the docked state of the GST molecule (point A). Proceeding to the solvated state, the Δ*G*_b_ smoothly decreases, displaying two main intermediate inflection points corresponding to two intermediate states, that is, one of the partially docked conformations (point B) and the partially solvated states (point C) of the G6 molecule, respectively. As expected, an almost constant Δ*G*_b_ value is observed when the GST molecule is fully solvated, because of the energetically equivalent conformations adopted by the solvated GST molecule (point D). To identify the x* discriminating the bound and unbound states of the system, we visually inspected the plot showing the Δ*G*_b_ changing the x* frame, and we picked the frame corresponding to the first inflection point from the bound state showing the ligand partially undocked from the binding site (point B in Figure 5). This criterion is similar in spirit to the “elbow criterion” used in machine learning for selecting the “right” number of clusters in k-means. The physical meaningfulness of x* is also evaluated. Moreover, the relative position of x* is further cross-checked versus the free energy profile. Indeed, the picked x* has also to be compatible with a transition-state-like nature on the PMF (see Figure 8a in the Discussion). If these checks fail, in the general case, we search for the following inflection point until these criteria are satisfied. At this stage, this procedure is still manually curated, and further work is needed to render it completely programmatic. Once x* is identified and checked against the qualitative and quantitative criteria, the probability ratio between the docked and solvated states of the ligand and the binding free energy, Δ*G*_b_, can be computed (eqs 5 and 6). In the Discussion section, we compare the result obtained for CB8-G6 with another CB8 complex showing a different behavior of Δ*G*_b_ in terms of x*, thus further discussing the strategy used to identify the x* frame, because this aspect is critical for the final result.

#### 3.2.2 Prioritization of the HST–GST Data Set on the Standard Binding Free Energy

**Figure 6:**
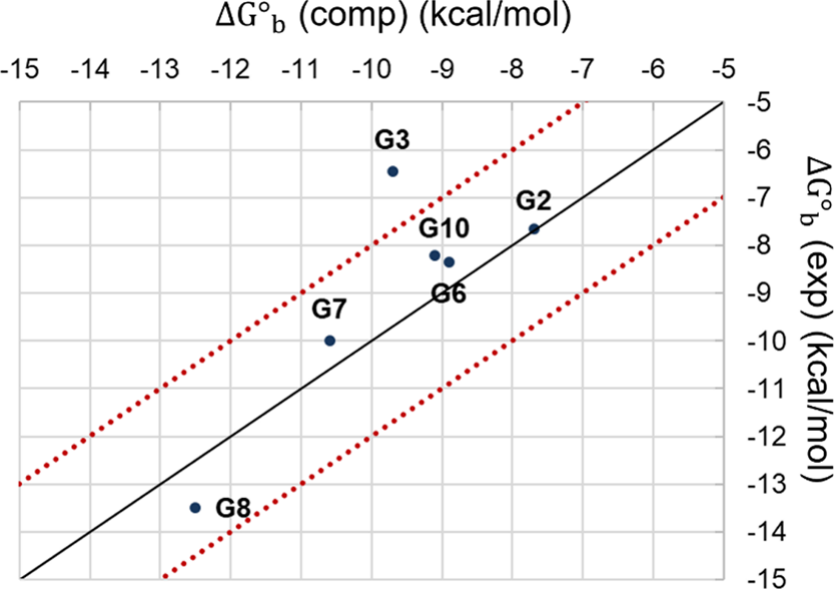
Scatter plot showing the experimental measurements for the HST–GST data set against the affinity predictions. The two red lines delimit the area within 2 kcal/mol from the diagonal (black line).

Table 1 and Figure 6 report the prioritization of the GST molecules on the standard binding free energy resulting from the WT-MetaD simulations and the isothermal titration calorimetry (ITC) measurements released for the SAMPL6 challenge.^43^ Each free energy term is labeled according to eq 5. The predicted standard binding free energy, Δ*G*^°^_b_, is reported together with the corresponding standard error, namely, the rate between the standard deviation and the square root of the sample size. With respect to the two asymmetric GST molecules (i.e., G2 and G3), the Δ*G*^°^_b_ values are reported as the average of the binding free energy values of both the exit directions, with the details for both exit directions indicated in the Supporting Information. We evaluated the Pearson correlation coefficient as a measure of the statistical relationship between experimental and computational estimates. From the data set in Table 1, it was equal to 0.84. By running a bootstrap resampling procedure, we assessed the statistical robustness of the correlation. Considering 10,000 bootstrap samples, we obtained an average Pearson correlation coefficient equal to 0.72 ± 4e-5 (i.e., the bootstrap standard error), confirming a good correlation between experimental and computational data. The Spearman coefficient was computed to assess the consistency of the prioritization of the binding affinities from computations and experiments (Spearman coefficient: 0.6), because absolute values are important, but ranking is probably even more cogent in drug discovery campaigns. The data were analyzed according to ref (66). The root mean square error (RMSE) of the predicted binding free energy values with respect to the experimental results is 1.5 kcal/mol. It is worth mentioning that we selected a limited number of GST molecules from the original data set presented in the SAMPL6 challenge. Thus, it is difficult to provide a comprehensive comparison of the performance of our method relative to those of the SAMPL6 challenge reported in ref (43).

The computational ranking of the CB8 series reported in Table 1 is in fairly good agreement with the experimental one, although with some relevant deviations (see G3, G6, and G10). However, G6 and G10 differ in experimental binding free energy by less than 0.2 kcal/mol, a quantity challenging to predict by computational methods and often within the experimental error. As reported in Table 1, the computational data tend to overestimate the binding free energy (mean error, ME: 0.7 kcal/mol), except for G8. This observation is not surprising, according to the SAMPL6 results obtained by applying methods relying on empirical force fields (i.e., GAFF) to predict the binding free energies of the HST–GST complexes.^43^ The accuracy between the computational and experimental datasets reported in Table 1 is around 1 kcal/mol for all the GST molecules except G3 for which the predicted and experimental Δ*G*^°^_b_ values differ by more than 3 kcal/mol. This deviation might be due to several aspects, such as G3’s large chemical structure making convergence more difficult.^67^ G3 might also have access to a second probable protonation state in water at the experimental pH,^43^ affecting the CB8-G3 binding affinity. Nevertheless, our binding free energy estimate for CB8-G3 is in line with previous computational results relying on other sampling methods, for example, double decoupling method^70^ and umbrella sampling^47,70^ that systematically overestimated the CB8-G3 binding affinity. Our approach reproduced the result for the CB8-G3 complex (Supporting Information) previously reported by Sun et al.^67^ Here, the authors identified the presence of multiple free energy minima corresponding to stable bound states of the G3 molecule in complex with CB8. This result was obtained using WT-MetaD to sample the spherical coordinates, ρ, θ, and φ. By validating this challenging result against the application of different computational protocols to explore diverse CVs, we increased our confidence in the predictions obtained for the benchmark data set. In addition, we ensured their independence from the starting configuration of the system and the peculiar dissociation path generated by an arbitrary enhanced sampling technique. In the Discussion section, we report additional considerations for the test case of CB8 in complex with the asymmetric GST molecules (G2 and G3). In the Supporting Information, we report all the PMFs, the Δ*G*^°^_b_ as a function of the x*, and the significant plots assessing the convergence of the WT-MetaD simulations for all the CB8 systems.

### 3.3 GSK-3β Kinase System

We then applied the same protocol to a real case study of pharmaceutical interest, the protein kinase GSK-3β. The unbinding kinetics of a strictly congeneric chemical series of pyrazine derivatives was previously characterized by both experiments and computations (i.e., elABMD). Here, we complete the characterization of those dissociation paths determined via elABMD by computing the underlying free energy profiles, referring to the thermodynamic experimental data reported by Berg et al.^44^ The chemical structures of the selected ATP-competitive inhibitors of GSK-3β are reported in Table 2. Compounds **1** and **4** display two positive and zero charges, respectively. For the remaining inhibitors in the series, one positive charge was assigned to the nitrogen of the 4-methylpiperazine group (R^1^).

**Table 2:**
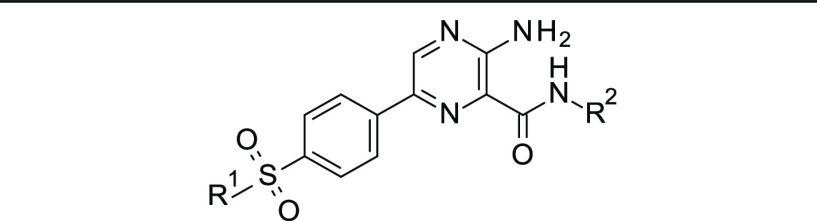

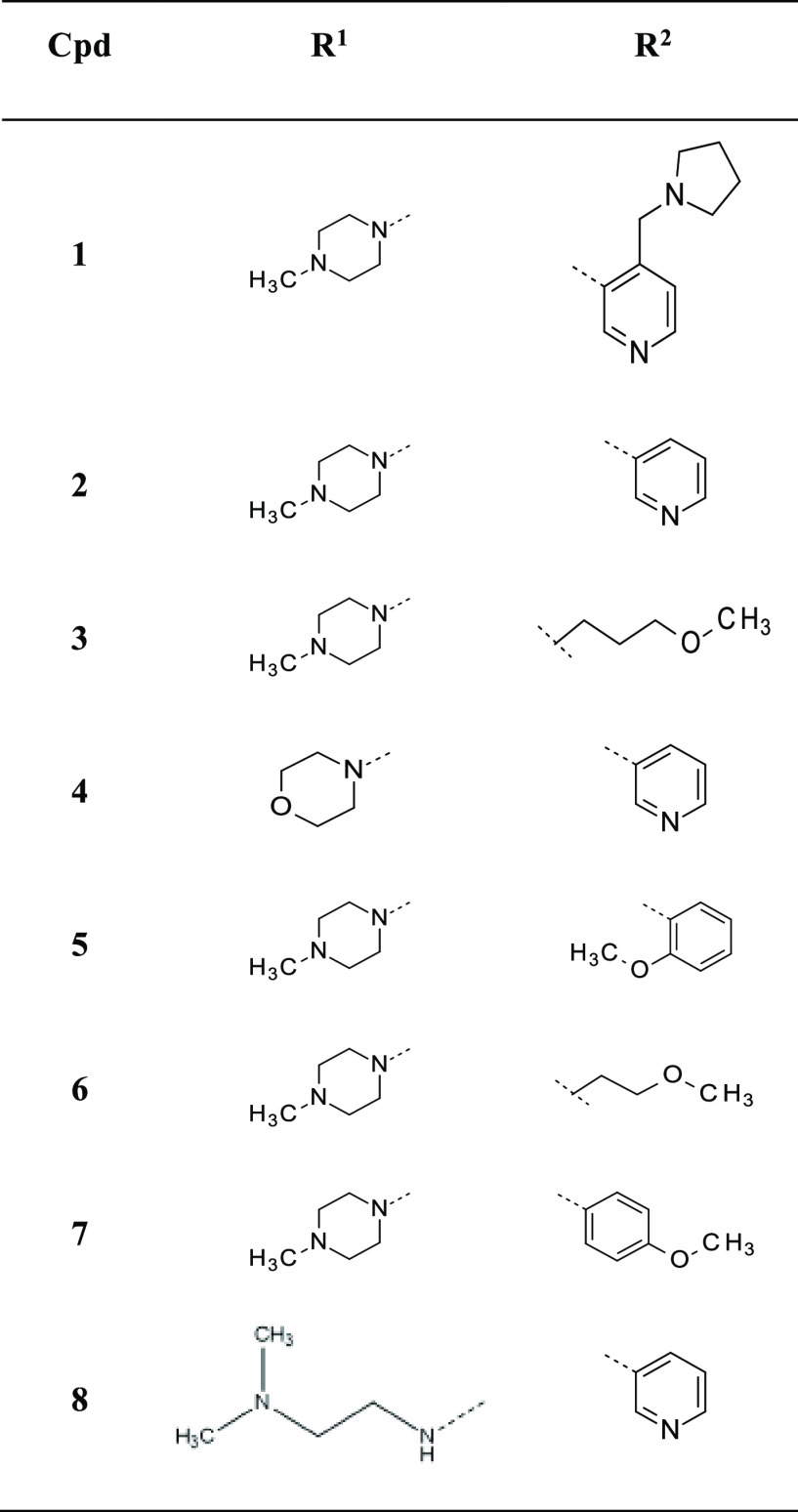
Chemical Structures of the Selected GSK-3β Inhibitors (**1**–**8**)

### 3.4 Prioritization of the GSK-3β Data set on the Standard Binding Free Energy

**Figure 7:**
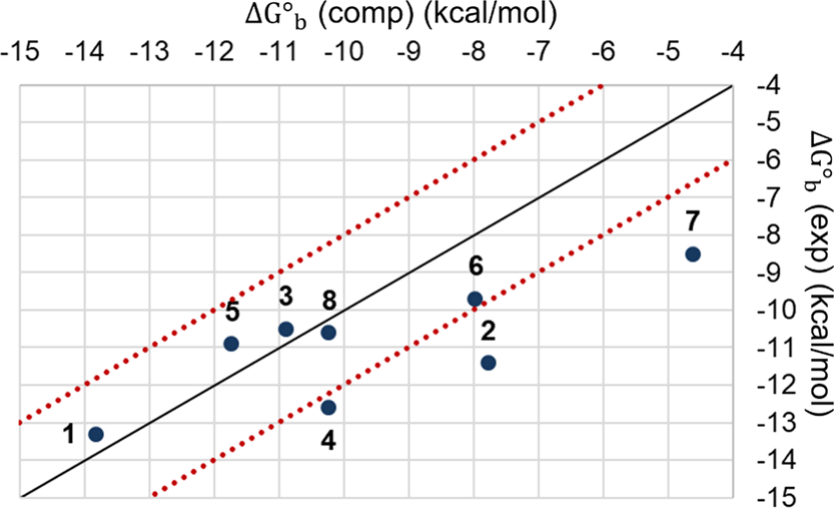
Scatter plot showing the experimental measurements for the GSK-3β series against the affinity predictions. The two red lines delimit the area within 2 kcal/mol from the diagonal (black line).

The dissociation paths for the GSK-3β complexes were generated via adiabatic bias MD coupled with an electrostatics-based CV, which provides a realistic description of the unbinding processes while sampling the metastable states of the system. This is particularly relevant in this kind of application because we observed that the proper sampling of the intermediate configurations facilitates the identification of the x* frame, thus leading to more reliable binding free energy estimates (see Section 4). Once the principal path was identified and optimized in terms of spacing between consecutive frames, we collected 1 μs of WT-MetaD along the PCVs for each GSK-3β complex and assessed the convergence of the WT-MetaD simulations as reported for the CB8 systems (Supporting Information). Table 3 and Figure 7 show the prioritization of the GSK-3β chemical series on the standard binding free energies. The Pearson correlation coefficient between computational and experimental values for the GSK-3β data set was 0.78. The bootstrapped estimate of the correlation coefficient was 0.70 ± 3e-5 (bootstrap standard error, 10,000 samples). In absolute terms, the most critical cases in the data set are **2** and **7** followed by **4** and **6**, further analyzed in the Discussion section. Consequently, the ranking of the GSK-3β series is not excellent, even though we generally observed a good agreement between computations and experiments (Spearman coefficient: 0.6). The RMSE for the predicted binding free energies relative to the experimental values is 2.2 kcal/mol; the ME results are equal to −1.3 kcal/mol suggesting the general trend toward underestimating the experimental binding free energies.

**Table 3:** Prioritization of the GSK-3β Inhibitors on Standard Binding Free Energies Obtained by WT-MetaD and Experiments^a^

CPD ID	Δ*G*_b_	Δ*G*_V_	Δ*G*^°^_b_	Rank_comp_	Δ*G*^°^_b,exp_	Rank_exp_
**1**	–13.6	–0.2	–13.8 ± 0.1	1	–13.3	1
**2**	–7.1	–0.7	–7.8 ± 0.3	7	–11.4	3
**3**	–10.4	–0.5	–10.9 ± 0.0	3	–10.5	6
**4**	–10.1	–0.1	–10.2 ± 0.2	4	–12.6	2
**5**	–11.5	–0.2	–11.7 ± 0.1	2	–10.9	4
**6**	–7.6	–0.3	–8.0 ± 0.1	6	–9.7	7
**7**	–4.2	–0.4	–4.6 ± 0.8	8	–8.5	8
**8**	–9.9	–0.3	–10.2 ± 0.9	5	–10.6	5

a The free energy terms (i.e., Δ*G*_b,_ Δ*G*_V_, Δ*G*^°^_b_, and Δ*G*^°^_b,exp_) are reported in kcal/mol. Pearson correlation coefficient: 0.78. Bootstrap Pearson correlation coefficient: 0.70 ± 3e-5 (bootstrap standard error, 10,000 samples). Spearman coefficient: 0.6. RMSE: 2.2 kcal/mol. ME: −1.3 kcal/mol. The experimental data refer to Berg et al.^44^

## 4 Discussion and Conclusions

**Figure 8:**
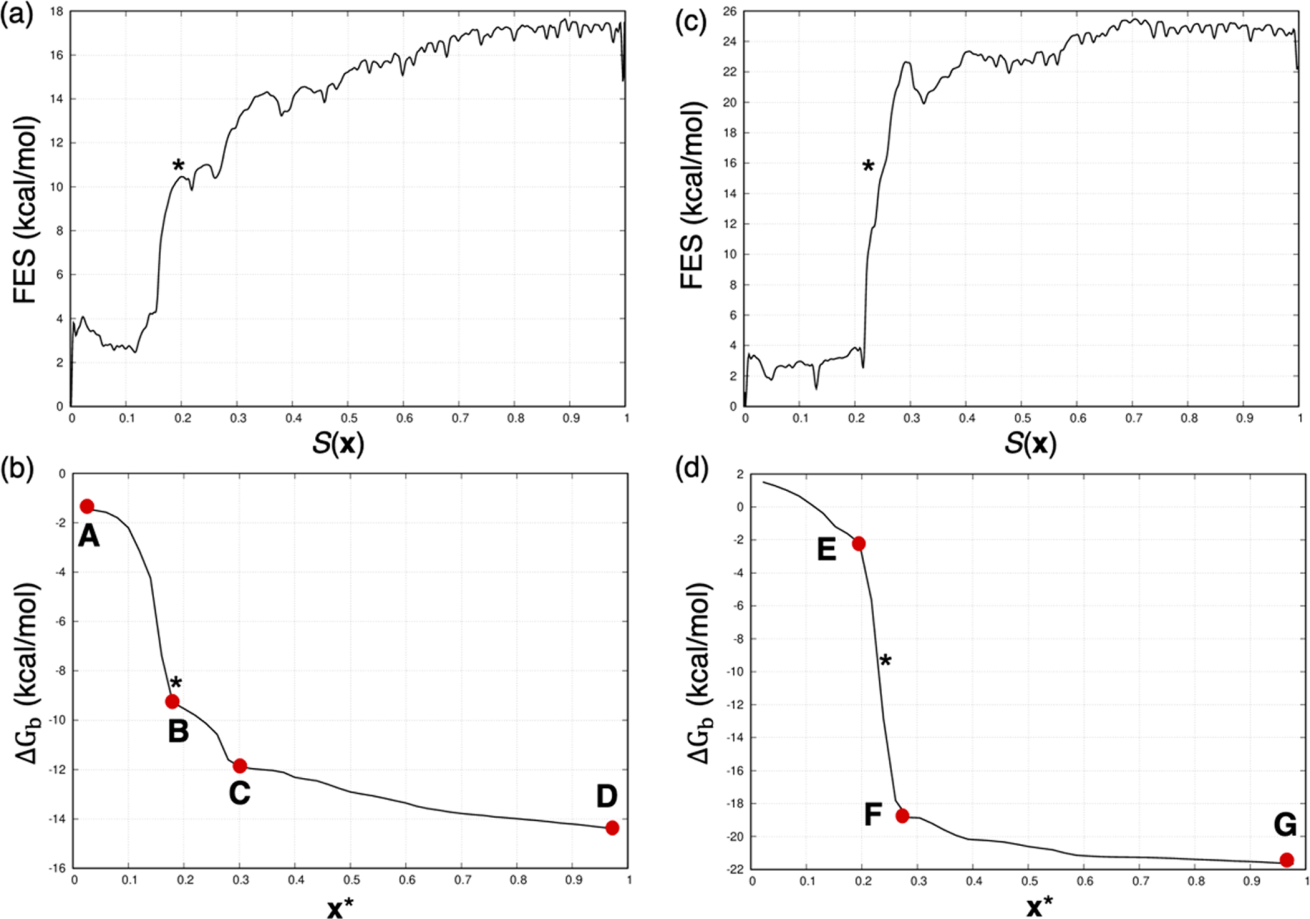
Free energy profiles and evolution of the binding free energy, Δ*G*_b_, changing the x* frame for the CB8-G6 (a and b) and the CB8-G8 (c and d) systems. For CB8-G6, the x* frame corresponds to the first inflection point encountered moving from the bound state (b, point B). In the PMF (a), it identifies the first energy barrier. For CB8-G8, because only one inflection point (d, point F) is observed between the bound (d, point E) and unbound (d, point G) states of the system, the x* frame is fixed between the two well-defined inflection points (d, points E and F). In this case, in the PMF (c), the x* frame corresponds to a not-sampled intermediate state between the lowest energy minimum (bound state) and the plateau corresponding to the solvated state of the system.

This study devises a semiautomated approach, of broad applicability, used here to compute the PMF and the standard binding free energy for protein–ligand complexes. The efficient and accurate estimation of the binding free energy remains one of the major open issues in computational drug discovery. In our protocol, a critical step was identifying the *S*(***x***) frame that separated the bound from the unbound states (here referred to as x*) to provide a realistic partition function and thus an accurate standard binding free energy estimation. We did not fully automatize the x* identification procedure (this is currently in progress), and we used a collection of cross-checked heuristics for analyzing the plot of the Δ*G*_b_ versus x*, the corresponding free energy profile, and the physical path. For the CB8-G6 case, x* corresponds to a configuration of the system showing the breaking of the key HST–GST contacts together with the GST molecule partially undocked from the binding site (Figure 5 and Figure 8b). The x* frame should, in principle, correspond to the transition state of the dissociation mechanism (Figure 8a), making our choice of x* similar to the one proposed in previous studies.^56,68^ It is worth mentioning that, even though our criterion to identify the x* is valid in most cases, there are systems in which some additional considerations may be required. For example, if the first inflection point from the bound state on the Δ*G*_b_ changing x* plot was not compatible with a reasonable free energy change in the free energy profile, we moved to the following one until a transition-state-like free energy barrier was found. This was the case of **4** reported in Section 4 in the Supporting Information. Here, the first inflection point at *S*(***x***) = 0.2 did not correspond to a significant free energy change, whereas this criterion was fulfilled considering the inflection point at *S*(***x***) = 0.4, which was picked as x* for this system. Another critical case is CB8-G8 investigated here. In Figure 8d, we report the Δ*G*_b_ for the CB8-G8 case, which shows a single fast intermediate inflection point (F) separating the docked (E) and solvated (G) states of the GST molecule. In these cases, we heuristically observed that the average point between two well-defined inflection points corresponding to the bound and unbound states (E and F in Figure 8d) is an acceptable approximation of the x* frame. We further highlight that identifying the x* frame is far from trivial, and it is difficult to detect it by looking at the free energy barriers along the PMF. Thus, human intervention is needed to validate the choice of the x* frame by visually inspecting the free energy path. Here, we observed that the steered MD protocol used to generate preliminary dissociation paths for all the HST–GST systems might not have properly sampled the intermediate metastable states for the CB8-G8 system. By failing to sample the transition state region between bound and unbound states, the principal path reconstructed for the CB8-G8 complex does not include any significant intermediate configurations that WT-MetaD can eventually sample. As such, the corresponding PMF is very steep, making it challenging to define a proper x* frame (Figure 8c). We emphasize that the x* frame identification was far simpler with GSK-3β, where we used adiabatic bias MD to generate preliminary dissociation paths.

To increase the GST molecules’ structural variability in the data set and to challenge our approach with noncongeneric compounds, two asymmetric GST molecules (G2 and G3) were also investigated. G2 and G3 also display the lowest binding affinities against the cucurbit[*n*]uril host molecule CB8, which determines the partial dissociation of the G2 and G3 GST molecules during NPT equilibration, before the steered MD simulation. As anticipated in the Section 2, steered MD was repeated twice for the CB8 in complex with the asymmetric G2 and G3 to generate dissociation paths involving both the exit directions. Concerning this point, we observed that both G2 and G3 display different PMF profiles, when the GST molecule unbinds in either exit direction, suggesting possible kinetic barriers driving the system toward the most energetically favorable dissociation path. As expected, the different free energy profiles for the asymmetric G2 and G3 lead to binding affinity predictions that differ by less than 1 kcal/mol, thus increasing our confidence in the computational estimates (see Table S1 in the Supporting Information).

**Figure 9:**
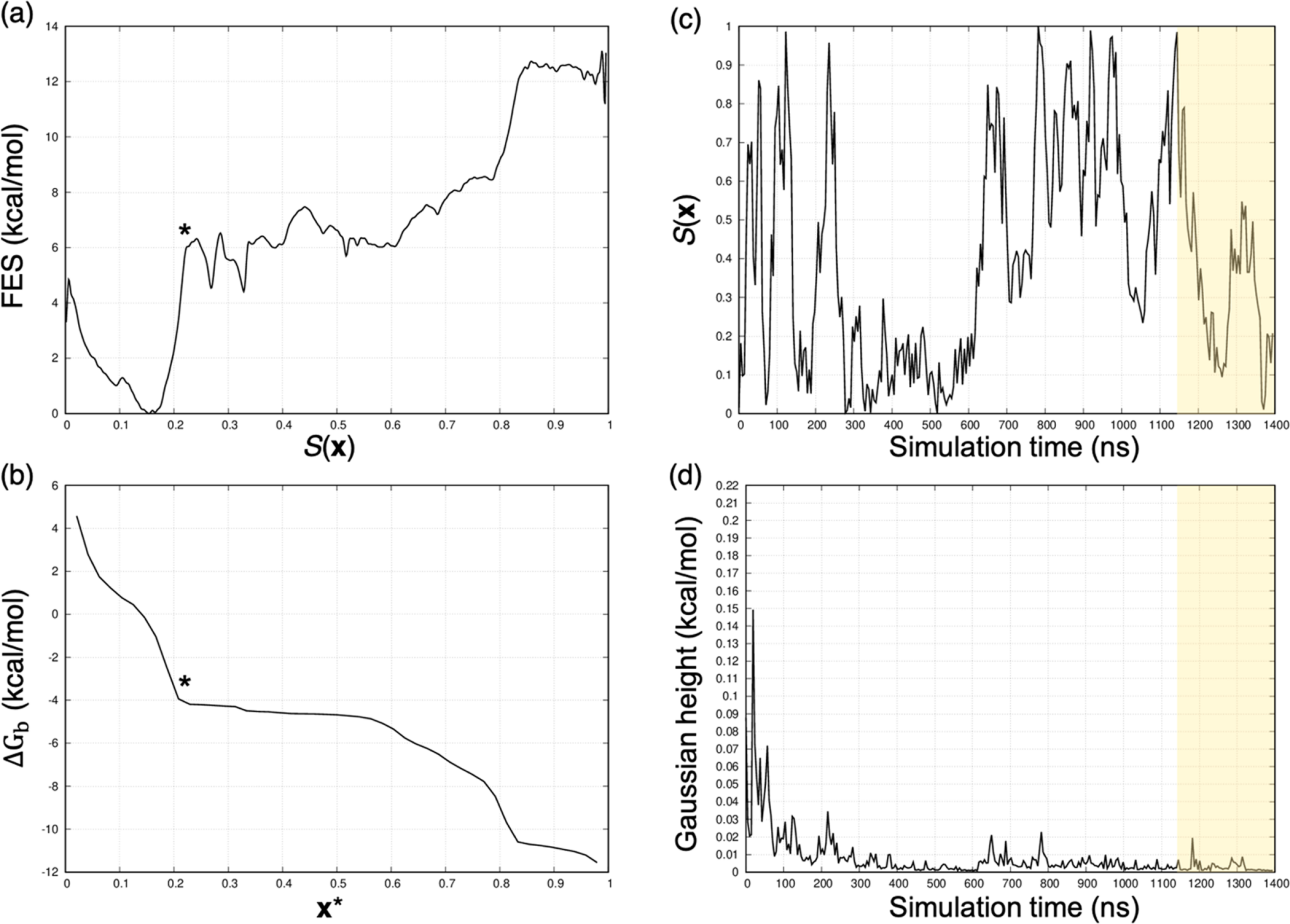
GSK-3β in complex with **7**. (a) Free energy profile and (b) identification of the x* frame. The behavior of (c) *S*(***x***) variable and (d) Gaussian hills against the simulation time. Looking at (c), we observe that the bound state (*S* = 0) for **7** is not properly sampled after 1 μs of WT-MetaD simulation. The shaded regions highlight the converged portion of the WT-MetaD simulation considered in the computation of the binding free energy reported in Table 3.

**Figure 10:**
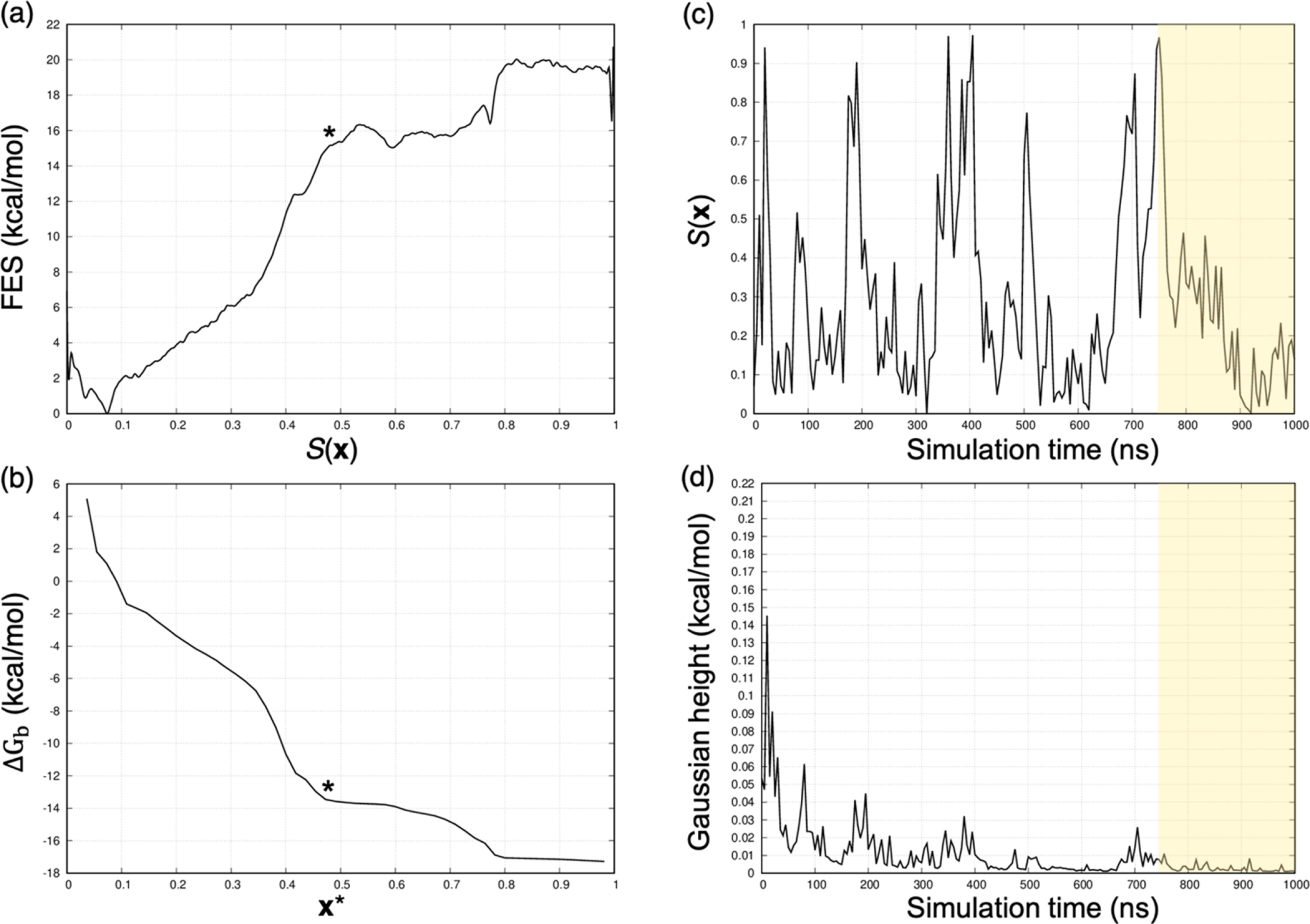
GSK-3β in complex with **1**. (a) Free energy profile and (b) identification of the x* frame. Behavior of (c) *S*(***x***) variable and (d) Gaussian hills against the simulation time. Panel (c) shows the diffusive behavior of the *S*(***x***) variable after 1 μs of WT-MetaD simulation. The shaded regions highlight the converged portion of the WT-MetaD simulation considered in the computation of the binding free energy reported in Table 3.

For the GSK-3β system, we evaluated the accuracy of the binding free energy estimations in absolute terms by comparing the computational estimate with the experimental reference for each complex. The most critical cases were **2** and **7** for which the free difference between the calculated and experimental values was more than 3.5 kcal/mol. By looking at the behavior of the *S*(***x***) variable, we observed that, after 1 μs of WT-MetaD simulation, the bound state of **7** was not correctly sampled (Figure 9c), possibly because of the suboptimal definition of the prior dissociation path. We would argue that the ABMD parameters that we selected for generating the dissociation paths with all the GSK-3β ligands were not appropriate for this compound. Indeed, we observed unphysical (high energy) ligand conformations during the unbinding event, probably because of the bias strength. We further highlight that the accuracy of the binding free energy estimates critically depends on how extensively the bound and prebound (intermediate) states have been sampled by WT-MetaD, as previously observed in several studies discussing binding free energy estimations for real systems. In light of this, we extended the WT-MetaD for **7** to 1.4 μs until a complete transition along the *S*(***x***) path was detected, and the height of the Gaussian hills was low to the point of not allowing further exploration of the *S*(***x***) path (Figure 9c,d). In Table 3, the computational free energy estimate for **7** refers to the extended simulation. In Figure 9a,b, the free energy profile for the dissociation of GSK-3β in complex with **7** is reported together with the plot representing the behavior of the Δ*G*_b_ against the x* frame. Notably, the validation of the computational result against the experimental data might be affected by the experimental low solubility of **7** observed in references (44, 50), thus questioning the accuracy of the experimental thermodynamic data reported in ref (44). For comparison, Figure 10 reports the GSK-3β/**1**, where standard binding free energy was estimated with high accuracy.

By evaluating the exploration of the *S*(***x***) path for **2**, we again observed that 1 μs of WT-MetaD simulation was not enough to let the system extensively explore the complex bound state. However, in contrast to **7**, the low height of the Gaussian hills for **2** meant we did not expect that proceeding further in the statistics would significantly change the exploration of the *S*(***x***) path (Supporting Information). As such, we did not extend the statistics for **2**. Moreover, the gap of approximately 2 kcal/mol observed for **4** and **6** was evaluated by looking at the simulation convergence. Similarly to **2**, we considered both simulations to have reasonably converged.

In conclusion, we introduced a semiautomated pipeline, which combines enhanced sampling simulations with a machine-learning method to predict standard binding free energies. As discussed by Mobley et al.,^69^ validated benchmark systems are crucial to understanding how different computational methods perform when attempting to compute the same thermodynamic properties. As such, we tested our workflow on one of the HST–GST systems suggested in the SAMPL6 challenge. Then, the method was applied to a system of pharmaceutical relevance, namely, GSK-3β/ligand complexes. In both cases, we obtained good binding affinity predictions in both relative and absolute terms. According to the present results, we were able to define the strengths and aspects that need to be improved to make this approach widely and routinely applicable to real drug discovery case studies. In particular, we would suggest using elABMD as an enhanced sampling technique to define the guess paths. This is because elABMD can provide an accurate description of the metastable conformations of the system when proper force constants are applied. A straightforward definition of the principal path, requiring minimum human intervention and negligible computational time, can then be obtained in combination with the equidistant waypoint algorithm, which prepares the path for WT-MetaD coupled with PCVs. Moreover, the system-independent procedure implemented to identify the x* frame allows one to obtain robust and accurate binding free energy estimates (through the partition function) provided that WT-MetaD simulations are converged. We are working on the automated identification of x* based on the finite difference approximation of the derivative of the PMF and a threshold free energy value. Finally, NanoShaper is a valuable tool for computing the sampling volume of the unbound state, thus allowing the accurate estimation of the binding free energy correction. The only step of the workflow that needs to be further investigated is the care in creating an “optimal” physical path that mimics a minimum free energy path as much as possible. Steered MD is inadequate, and elABMD proves much better. However, not all elABMD trajectories are “optimal”, and it is always desirable to increase the “gentleness” of ligand release. We mention here that predicting absolute values via path-based free energy methods is far more computationally expensive and challenging relative to alchemical approaches widely applied in pharmaceutical settings. As such, physical path-based methods may not necessarily be more effective from the drug discovery viewpoint if only a number, namely, the free energy difference, is desired. The amount of information one extracts from the full PMF is not comparable with the output of alchemical methods. In the next future, we aim to apply the computational workflow depicted in Figure 1 to characterize rare events involving other chemical processes somewhat more complex than ligand unbinding.

## Supporting Information Available

(1) A description of the input matrix to the path-finding algorithm; (2) more details regarding the NanoShaper-based strategy employed to quantify the unbound volume; (3) additional computational data regarding the asymmetric GST molecules, G2 and G3, with relative plots; (4) for all the compounds not included in the main text, the plots representing: the free energy profiles, the Δ*G*_b_ against the ***x**** frame, variation of *S*(***x***), and the WT-MetaD Gaussian height along the simulation time; (5) plots representing the variation of Δ*G*_b_ along the simulation time for all the investigated compounds; and (6) the pseudocode of the equidistant waypoints algorithm (PDF)
