# Supplementary material for: Machine Learning and Enhanced Sampling Simulations for Computing the Potential of Mean Force and Standard Binding Free Energy
Source: J Chem Theory Comput. 2021 Jul 14;17(8):5287–300. doi: 10.1021/acs.jctc.1c00177 (PMC8389529; doi:10.1021/acs.jctc.1c00177)
Supplement: Supplementary file 1 — ct1c00177_si_001.pdf [file ct1c00177_si_001.pdf]

Machine Learning and Enhanced Sampling Simulations for Computing the Potential of Mean Force and Standard Binding Free Energy

Authors

Martina Bertazzo[†1,2,‡], Dorothea Gobbo[†1], Sergio Decherchi*[1,3], Andrea Cavalli*[1,2]

[1]Computational and Chemical Biology, Fondazione Istituto Italiano di Tecnologia, via Morego 30, 16163 Genoa, Italy

[2]Department of Pharmacy and Biotechnology (FaBiT), Alma Mater Studiorum – University of Bologna, via Belmeloro 6, 40126 Bologna, Italy

[3]BiKi Technologies s.r.l., Via XX Settembre 33/10, Genoa, Italy

[‡]Current affiliation: Global Research Informatics/Computational Chemistry, Evotec (France) SAS, 31100 Toulouse, France

†These authors have equally contributed to the work

*co-corresponding: sergio.decherchi@iit.it, andrea.cavalli@iit.it

1. Input matrix to the path finding algorithm

As reported in Section 2.2 in the Main Text, given a points cloud, the principal path algorithm connects two points defined a priori in data space and tries to pass through the local support of the data distribution, capturing the morphing path that passes through high probability regions. To compute the principal path through the regularized k-means algorithm (Equation 1 in the Main Text), an input $N \times d$ matrix needs to be calculated, where d holds the information on the coordinates in the 3D-dimensional space of the atoms selection and N is the total number of frames in the post-processed trajectory, X. The $N \times d$ matrix is defined with the coordinates of the heavy atoms of all the host-guest molecules included in the dataset. For the GSK-3β complexes, the $N \times d$ matrix is defined by the coordinates of the heavy atoms of the ligand and the protein residues located within a distance of 4 Å from the ligand in the bound state. Thus, every row of X reports the coordinates d in a specific frame of the trajectory.

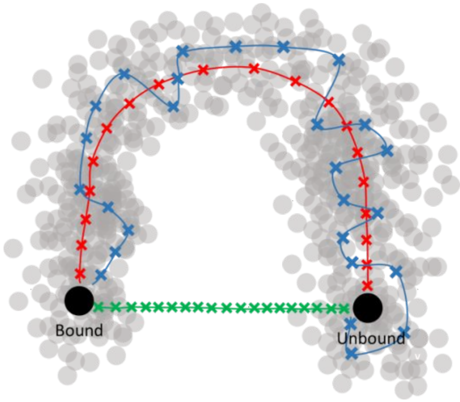

Figure S1. The hyper-parameter *s* influences the trade-off between the data fitting and the smoothness of the path. Data are represented as grey dots and waypoints as blue crosses. The irregular blue path refers to the setup where *s* value tends to zero showing a classical clustering solution. The green path refers to the setup where *s* value tends to infinity. The red path refers to the path found by properly choosing the value of the hyper-parameter *s*.

2. Nanoshaper-based strategy to quantify the unbound volume

As reported in Section 2.4 in the Main Text, we quantified the volume explored by the ligand in the unbound state by first identifying the frames in the path sampled by WT-MetaD showing the ligand in the bulk solvent. Then, we build a collective PDB that is the union of all the frames associated to the unbound state of the ligand; obviously we keep only the ligand selection in the PDB. On this artificial PDB we compute the SES which automatically delivers the volume traced by the ligand in the unbound state (Figure 4 in the Main Text). This provides an accurate approximation of the volume traced by the solvated ligand, V_{bulk}, needed to compute the volume correction of the binding free energy, as reported in Equation 5 in the Main Text. When computing the SES, we referred to the Amber radii parameterization that is given by default when using the BiKi Life Sciences software package[1] in which NanoShaper[2] is available. For details regarding NanoShaper we refer the reader to Ref.2.

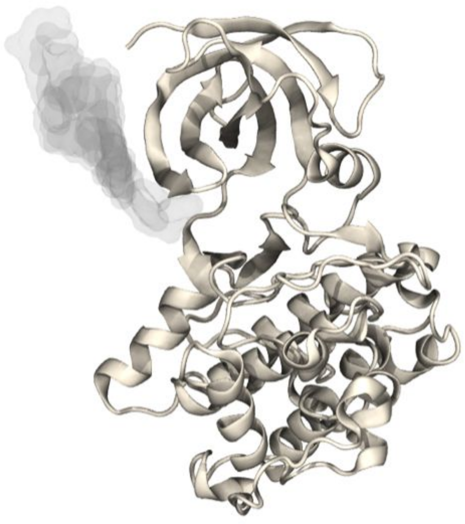

Figure S2. Solvent Excluded Surface (grey shaded volume) obtained by NanoShaper as the union of the ligand atoms concerning all the frames defining the unbound state of one representative GSK-3β complex.

3. Asymmetric guest molecules, G2 and G3

Given the top-bottom symmetry of the guest CB8, two asymmetric guest molecules, G2 and G3, have been included in the benchmark dataset. In Table S1, we report the estimated binding affinities for both exit directions together with the experimental data. Table 1 in the Main Text shows the average of the computed ΔG°_{b}.

Table S1. Standard binding free energies computed for both exit directions for G2 and G3[a]

GST ID	Exit direction	ΔG_b	ΔG_V	ΔG°_{b}	$\Delta G^{\circ}_{b,exp}$
G2	+1	-7.7	0.2	-7.5	-7.66 ± 0.05
	-1	-7.8	-0.2	-8.0	
G3	+1	-9.2	-0.2	-9.4	-6.45 ± 0.06
	-1	-10.0	0.0	-10.0	

[a] All the free energy terms (i.e. $\Delta G_{b,}$ ΔG_V, ΔG°_{b}, $\Delta G^{\circ}_{b,exp}$) are reported in kcal/mol.

In the following panels, (a) the free energy profiles, (b) the ΔG_b against $\mathbf{x}^*$, and the behaviour of (c) the S($\mathbf{x}$) variable and (d) the Gaussian hills along the simulation time are reported for G2 and G3 considering both the exit directions.

CB8-G2 (1)

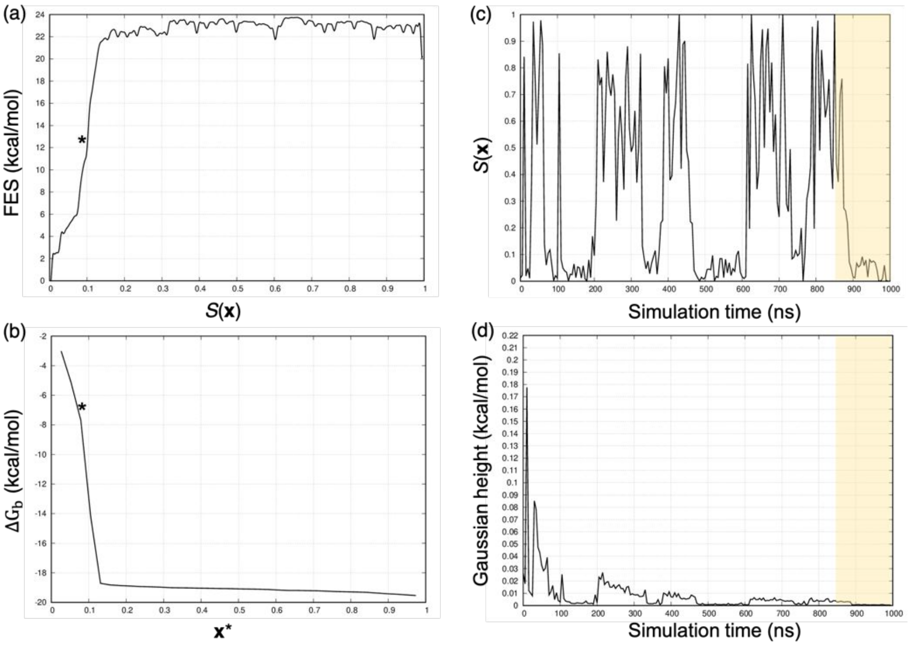

CB8-G2 (-1)

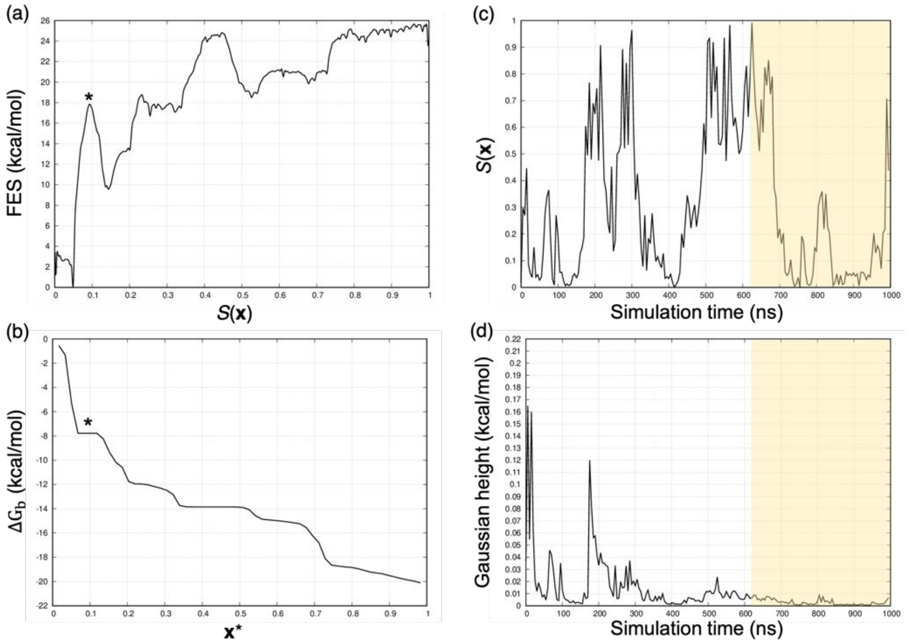

CB8-G3 (1)

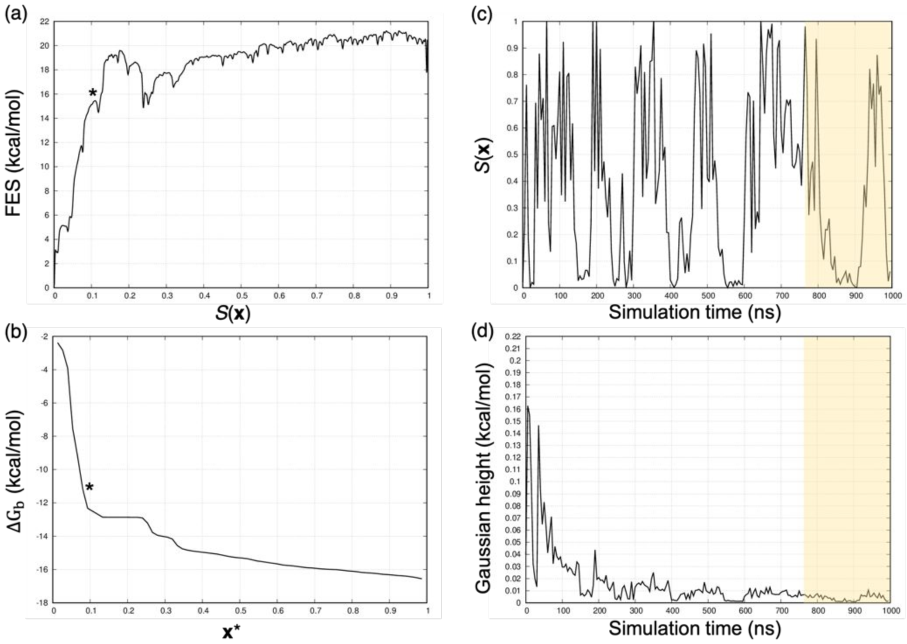

CB8-G3 (-1)

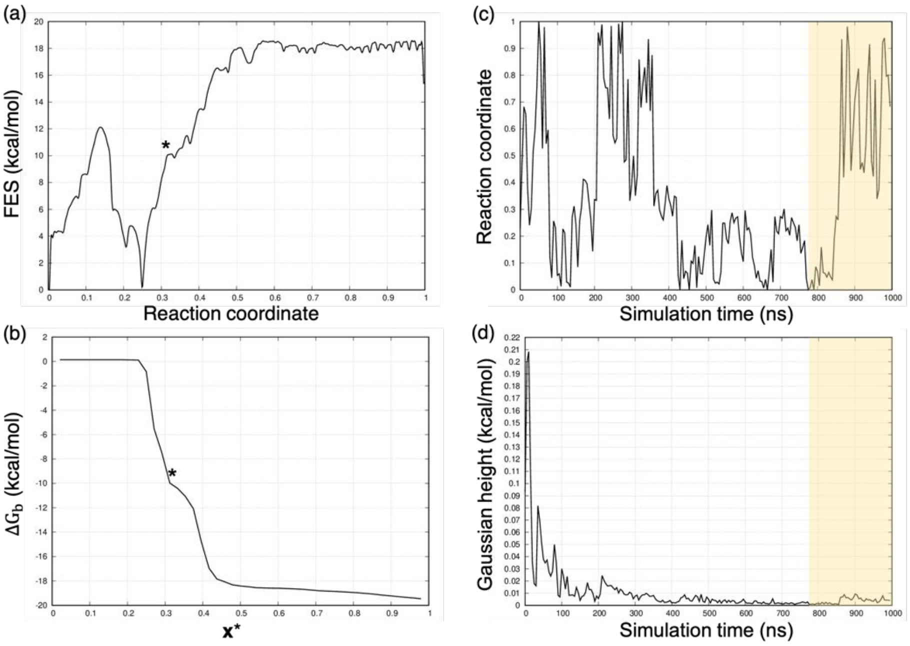

4. Free energy profiles, identification of the unbound frame (x*), variation of the S(x) variable and the Gaussian height along the simulation time

In the following panels we report (a) the free energy profiles, (b) the ΔG_b against $\mathbf{x}^*$, and the behaviour of (c) the S(**x**) variable and (d) the Gaussian hills along the simulation time for the following systems not reported in the Main Text: CB8-G7, CB8-G8, CB8-G10, and the GSK-3β complexes **2**, **3**, **4**, **5**, **6**, and **8**.

CB8-G7

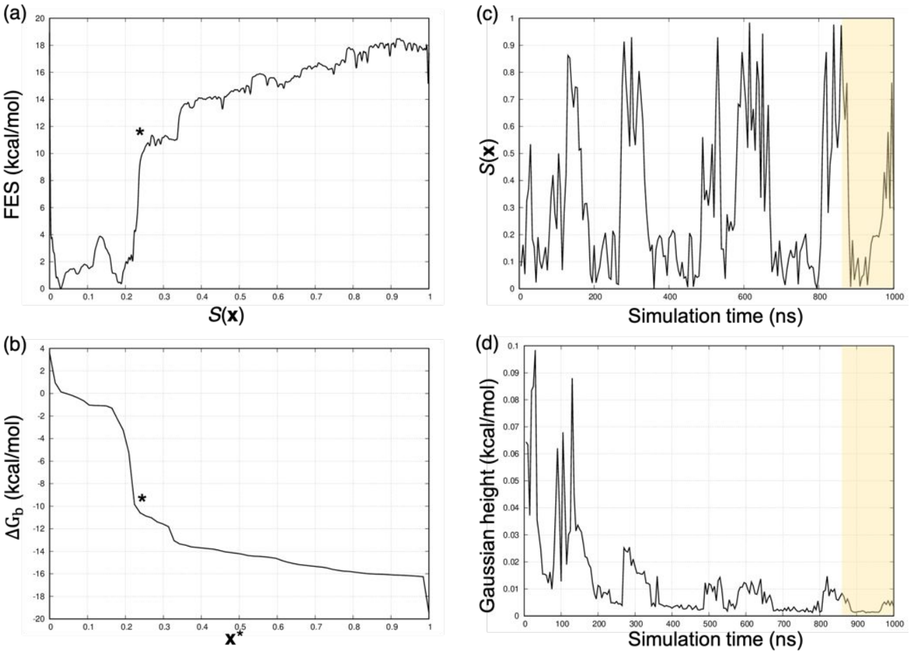

CB8-G8

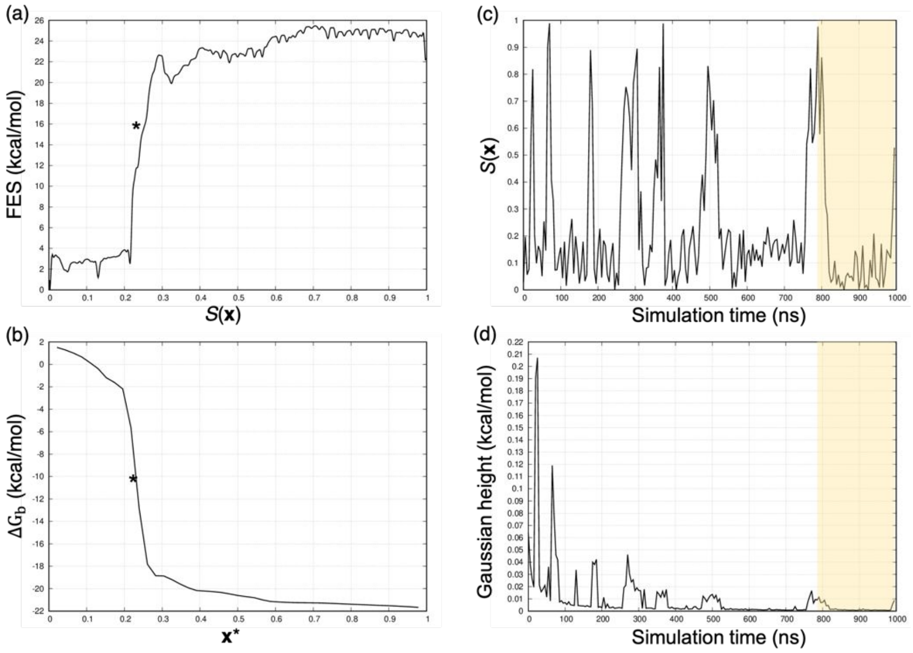

CB8-G10

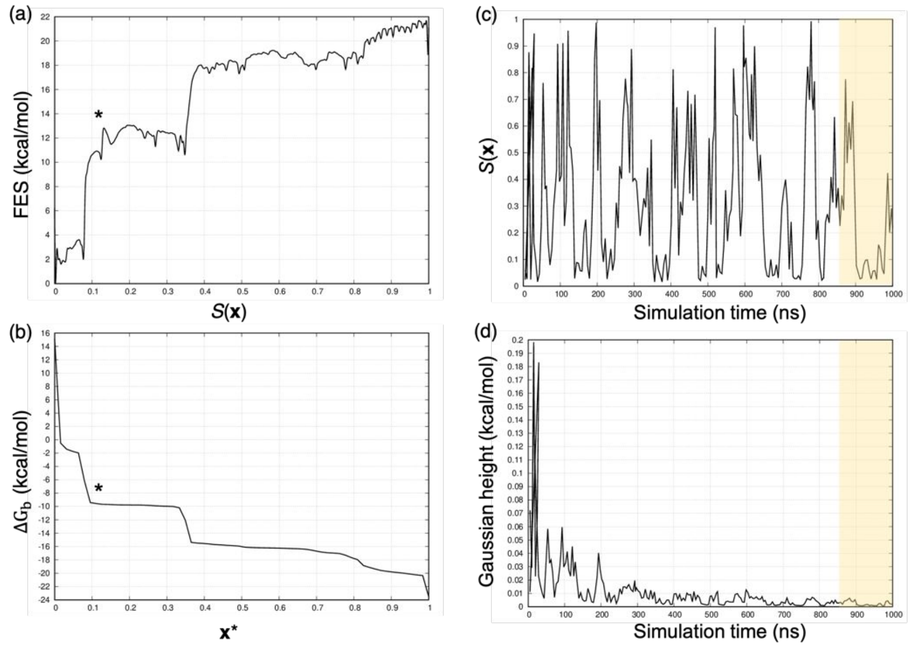

2

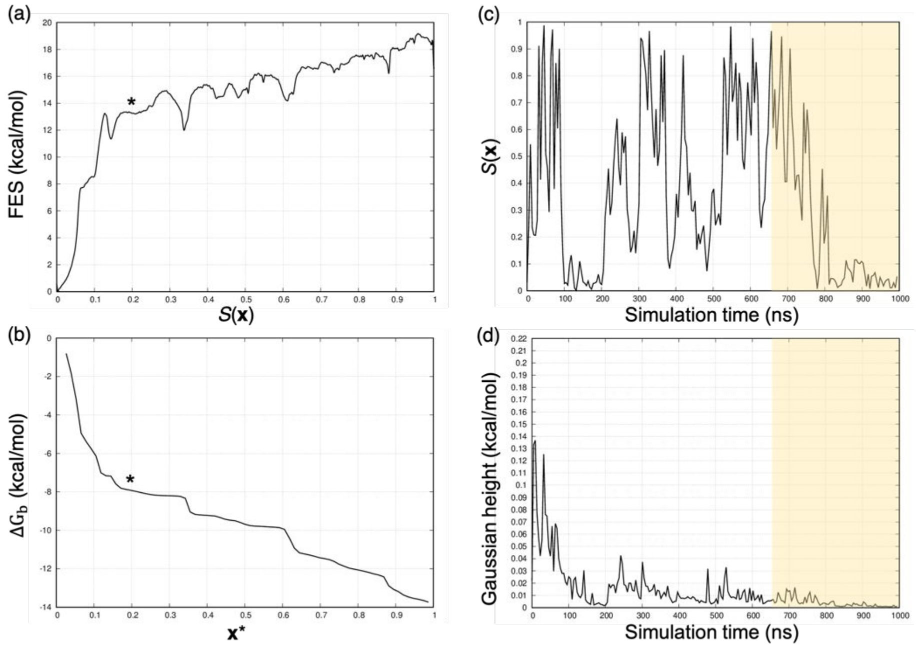
(a)
FES (kcal/mol)
S(x)
(b)
ΔG_b (kcal/mol)
x*
(c)
S(x)
Simulation time (ns)
(d)
Gaussian height (kcal/mol)
Simulation time (ns)

3

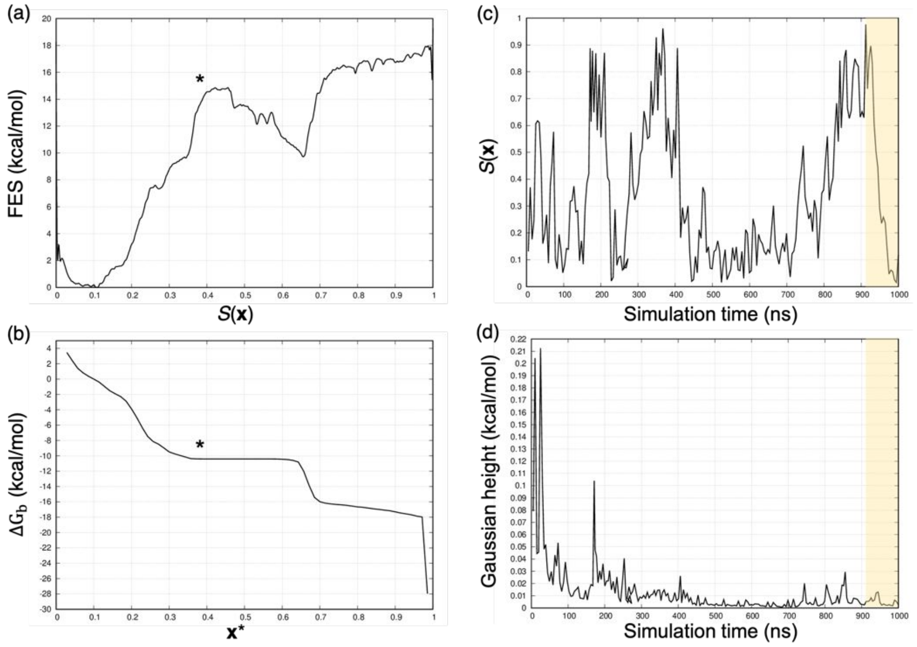
(a)
FES (kcal/mol)
S(x)
(b)
ΔG_b (kcal/mol)
x*
(c)
S(x)
Simulation time (ns)
(d)
Gaussian height (kcal/mol)
Simulation time (ns)

4

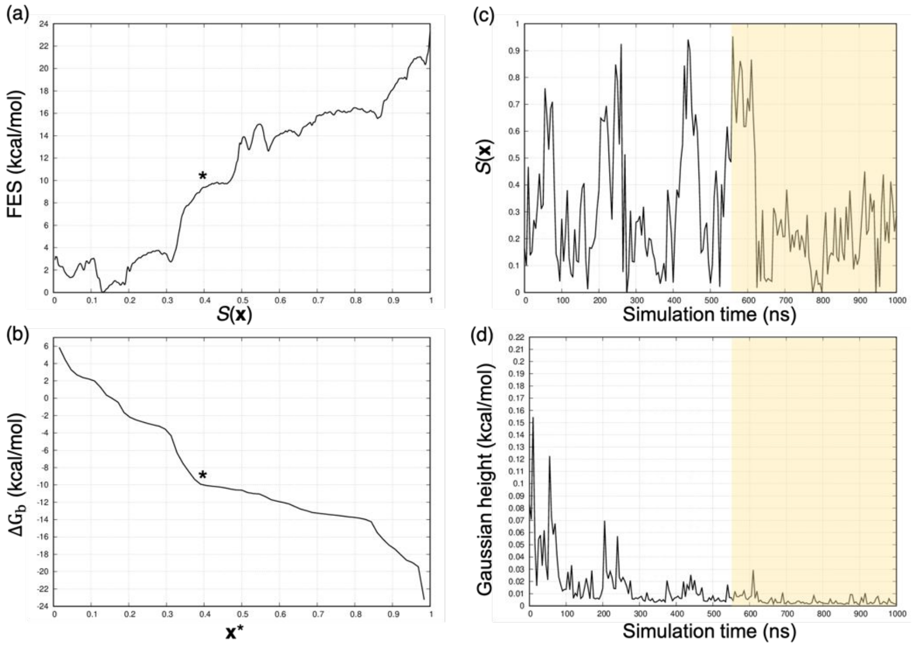
(a)
FES (kcal/mol)
S(x)
(b)
ΔGb (kcal/mol)
x*
(c)
S(x)
Simulation time (ns)
(d)
Gaussian height (kcal/mol)
Simulation time (ns)

5

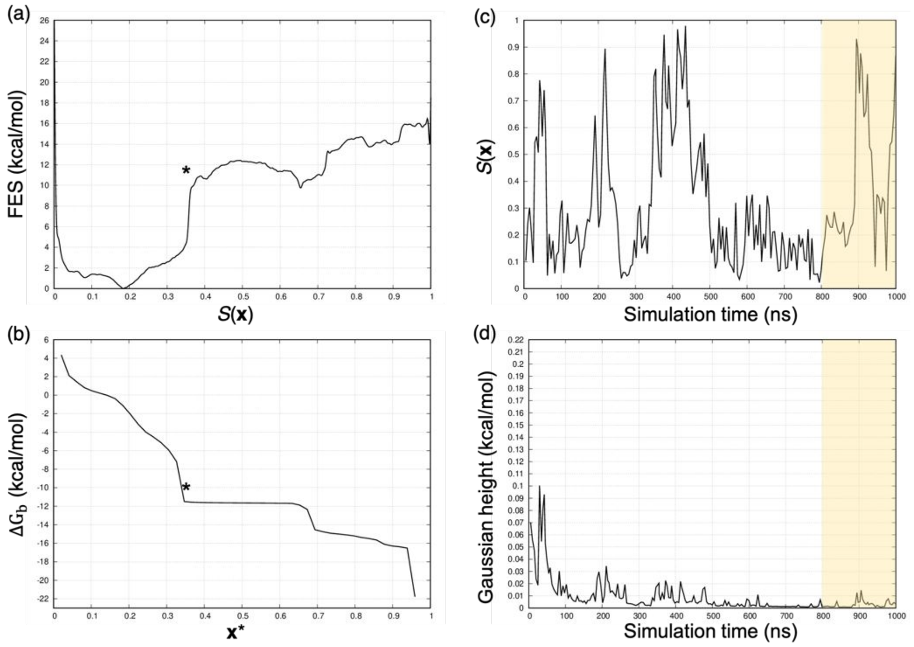
(a)
FES (kcal/mol)
S(x)
(b)
ΔGb (kcal/mol)
x*
(c)
S(x)
Simulation time (ns)
(d)
Gaussian height (kcal/mol)
Simulation time (ns)

6

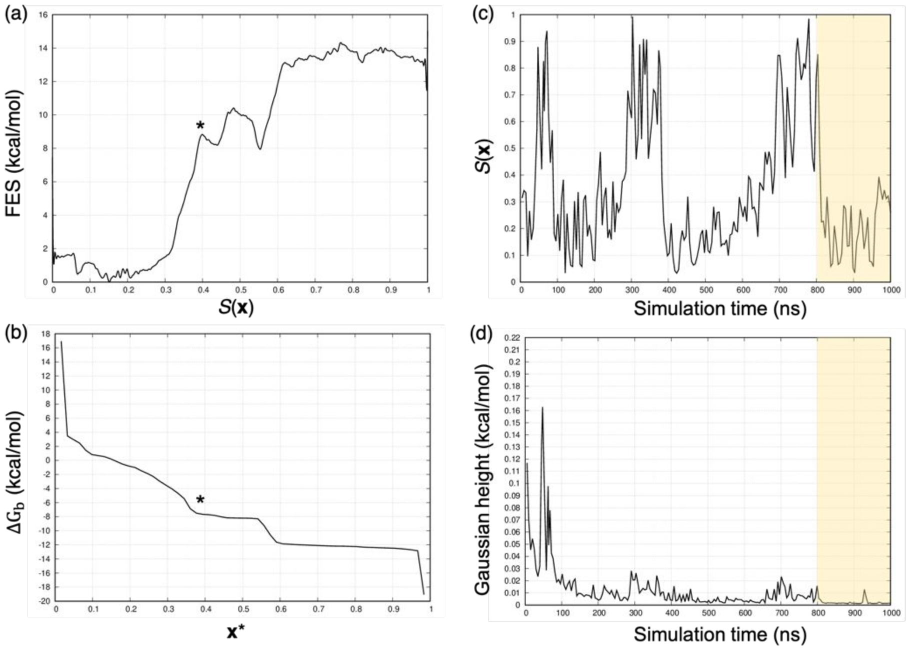

(a)
FES (kcal/mol)
S(x)
*
(b)
ΔG_b (kcal/mol)
x*
*
(c)
S(x)
Simulation time (ns)
(d)
Gaussian height (kcal/mol)
Simulation time (ns)

8

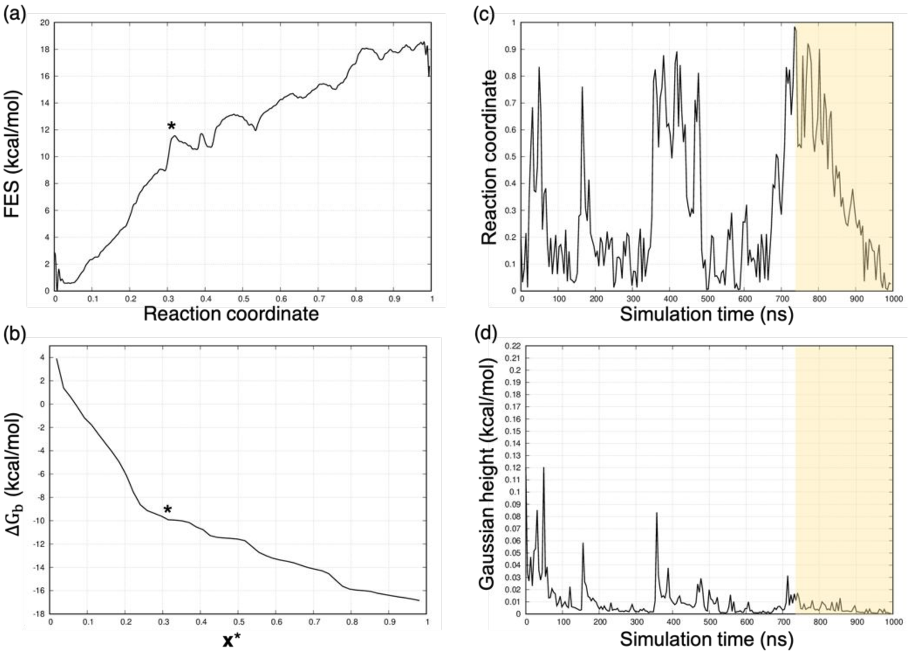

(a)
FES (kcal/mol)
Reaction coordinate
*
(b)
ΔG_b (kcal/mol)
x*
*
(c)
Reaction coordinate
Simulation time (ns)
(d)
Gaussian height (kcal/mol)
Simulation time (ns)

5. Binding free energy as a function of the simulation time

Considering that the temporal evolution of the binding free energy is a relevant quantity to assess the convergence of MetaD-based simulations, we report in the following panels the evolution of (c) the *S*(**x**) variable and (e) the binding free energy for all the systems. In this work, we assessed the convergence by looking at the temporal evolution of the *S*(**x**) variable and the Gaussian hills height, as reported in the Main Text, and ultimately we took into account the behaviour of the binding free energy. The shaded regions highlights the converged portion of the WT-MetaD simulations considered to estimate the binding free energies reported in the Main Text.

CB8-G2 (+1)

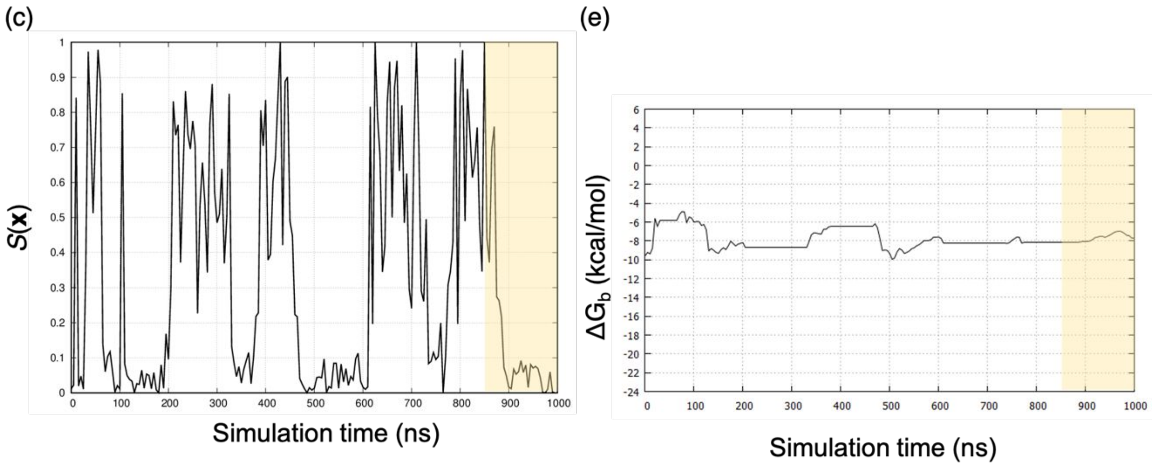

CB8-G2 (-1)

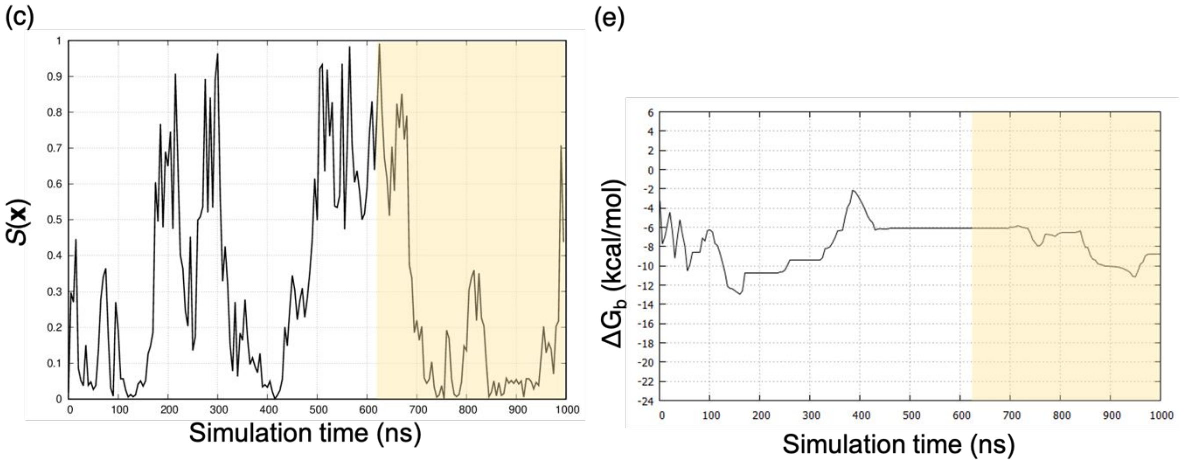

CB8-G3 (+1)

(c)

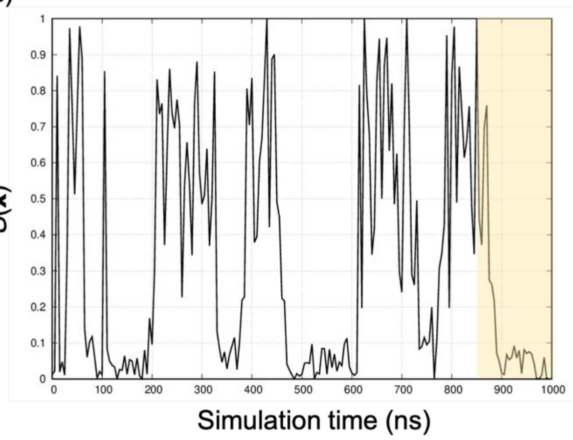

(e)

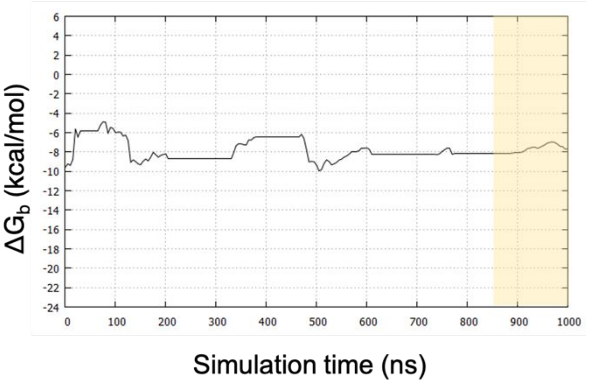

CB8-G3 (-1)

(c)

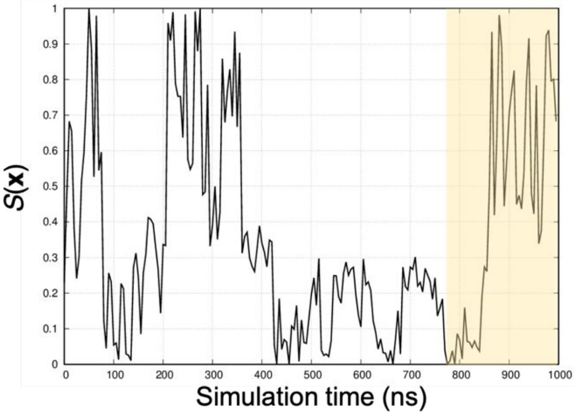

(e)

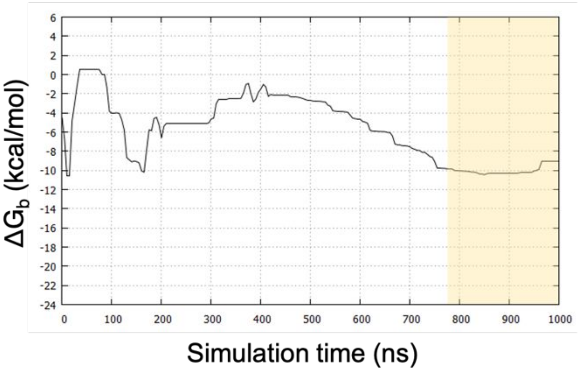

CB8-G6

(c)

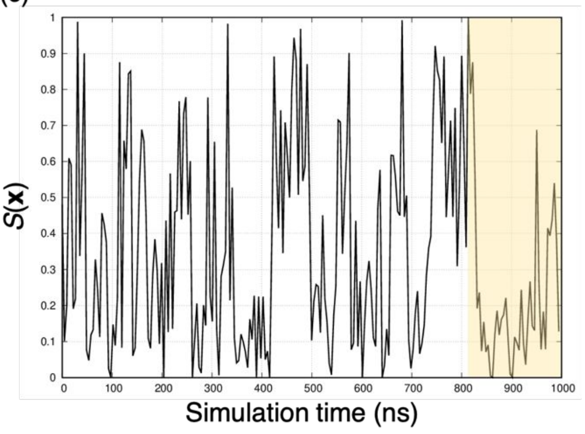

(e)

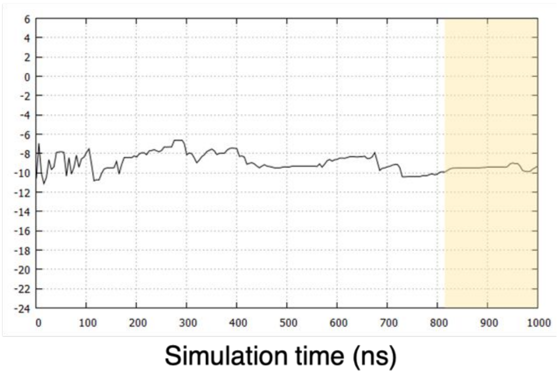

CB8-G7

(c)

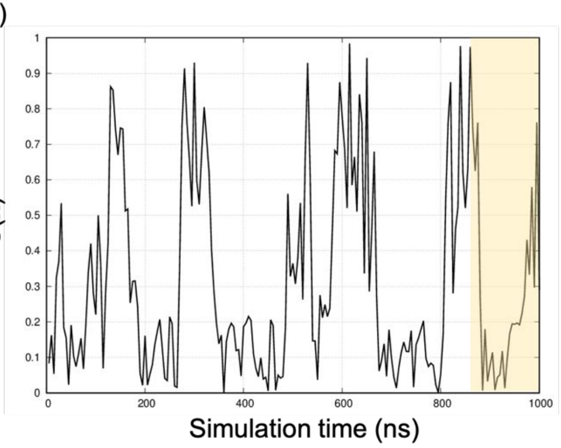

(e)

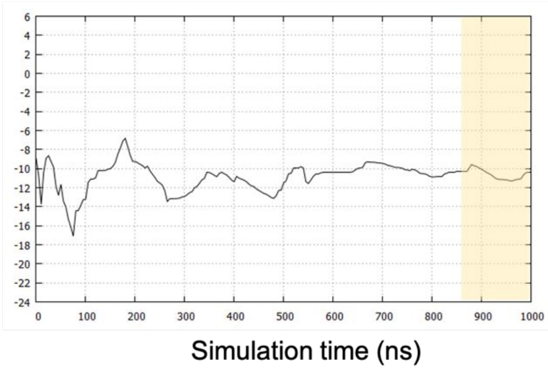

CB8-G8

(c)

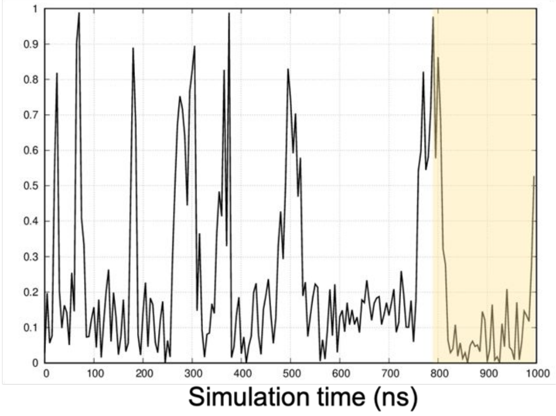

(e)

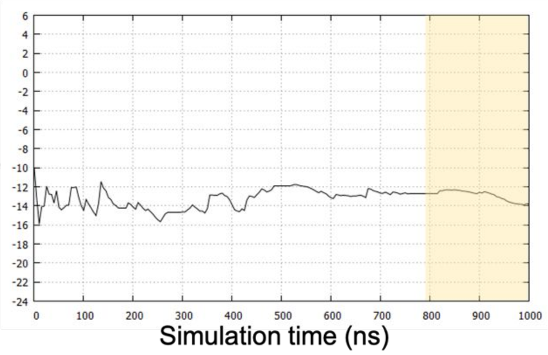

CB8-G10

(c)

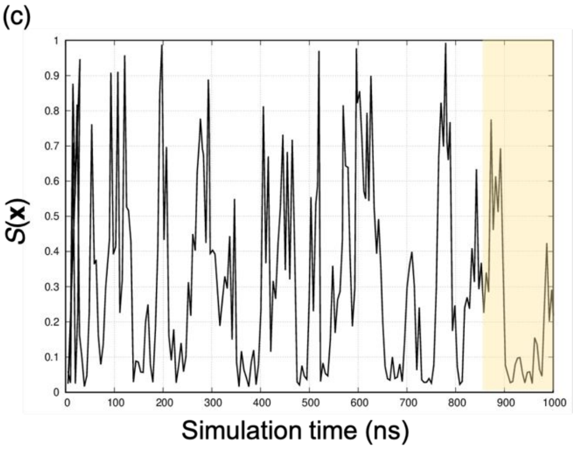

(e)

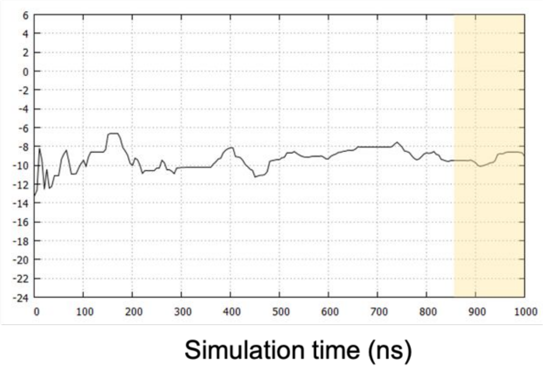

1

(c)

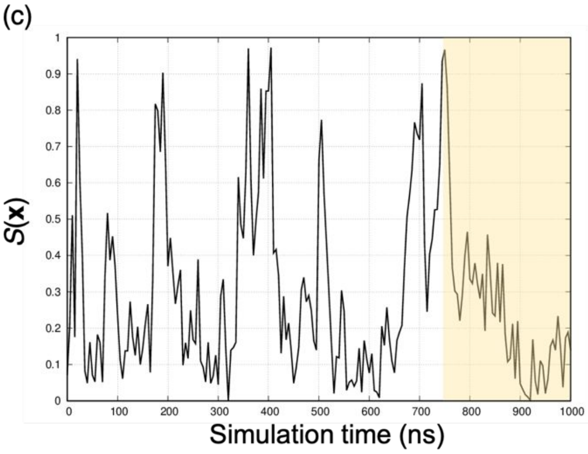
S(x)
0 0.1 0.2 0.3 0.4 0.5 0.6 0.7 0.8 0.9 1
0 100 200 300 400 500 600 700 800 900 1000
Simulation time (ns)

(e)

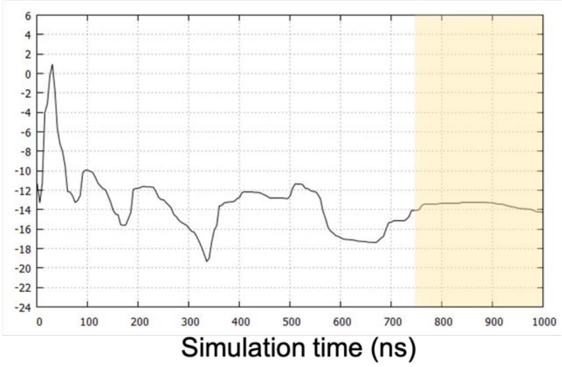
ΔG_b (kcal/mol)
6 4 2 0 -2 -4 -6 -8 -10 -12 -14 -16 -18 -20 -22 -24
0 100 200 300 400 500 600 700 800 900 1000
Simulation time (ns)

2

(c)

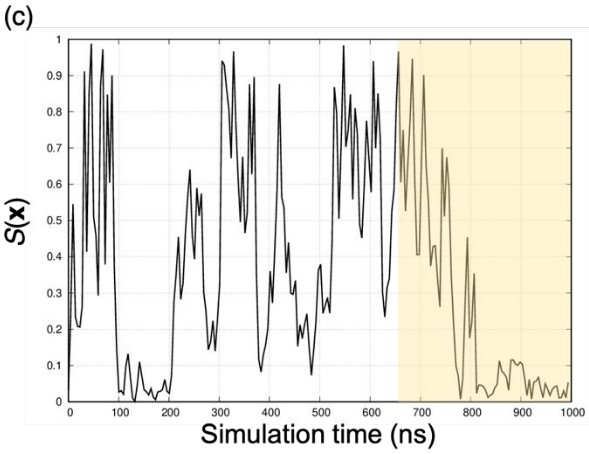
S(x)
0 0.1 0.2 0.3 0.4 0.5 0.6 0.7 0.8 0.9 1
0 100 200 300 400 500 600 700 800 900 1000
Simulation time (ns)

(e)

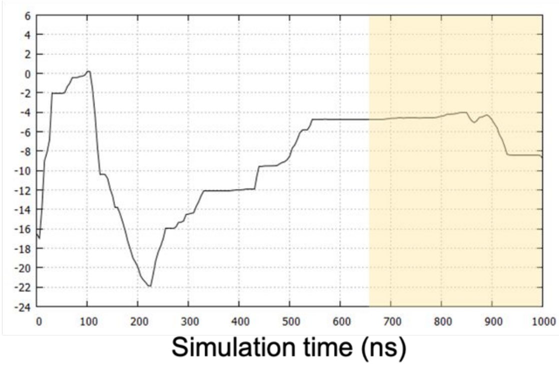
ΔG_b (kcal/mol)
6 4 2 0 -2 -4 -6 -8 -10 -12 -14 -16 -18 -20 -22 -24
0 100 200 300 400 500 600 700 800 900 1000
Simulation time (ns)

3

(c)

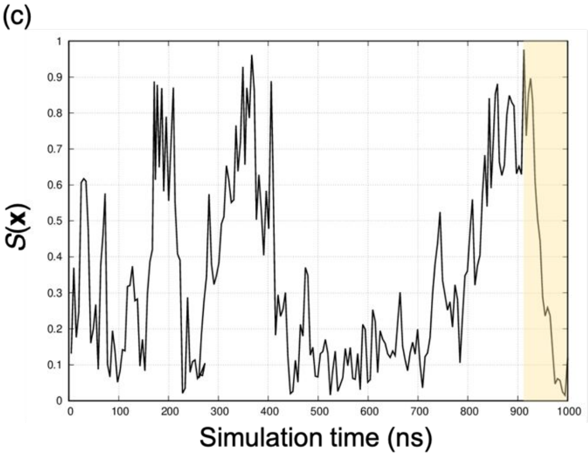
S(x)
0 0.1 0.2 0.3 0.4 0.5 0.6 0.7 0.8 0.9 1
0 100 200 300 400 500 600 700 800 900 1000
Simulation time (ns)

(e)

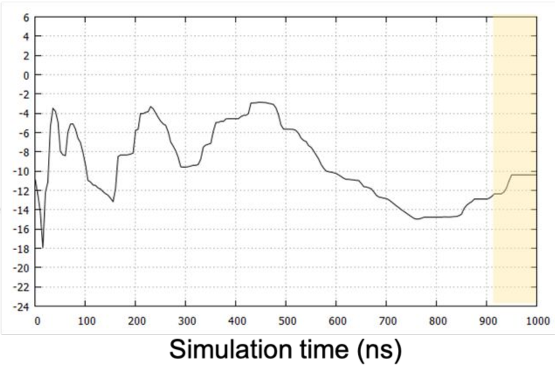
ΔG_b (kcal/mol)
6 4 2 0 -2 -4 -6 -8 -10 -12 -14 -16 -18 -20 -22 -24
0 100 200 300 400 500 600 700 800 900 1000
Simulation time (ns)

4

(c)

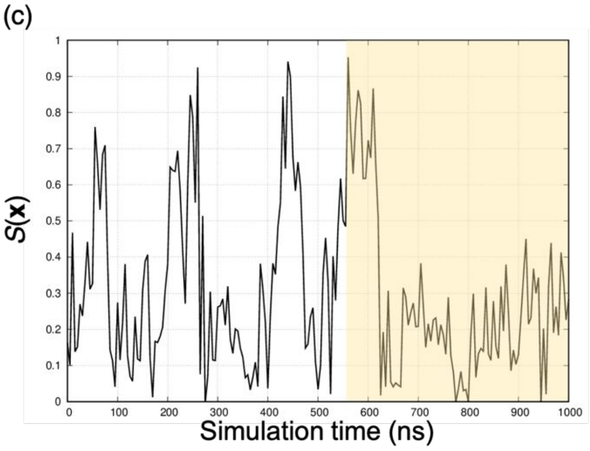
S(x)
Simulation time (ns)

(e)

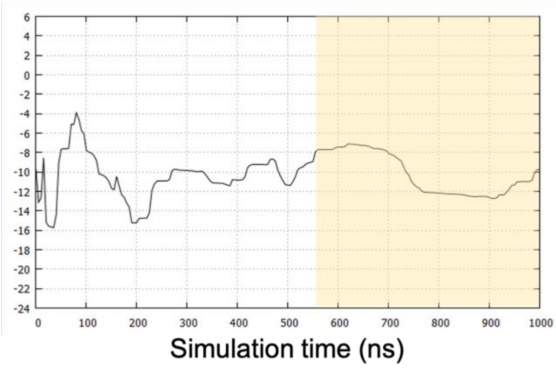
ΔG_b (kcal/mol)
Simulation time (ns)

5

(c)

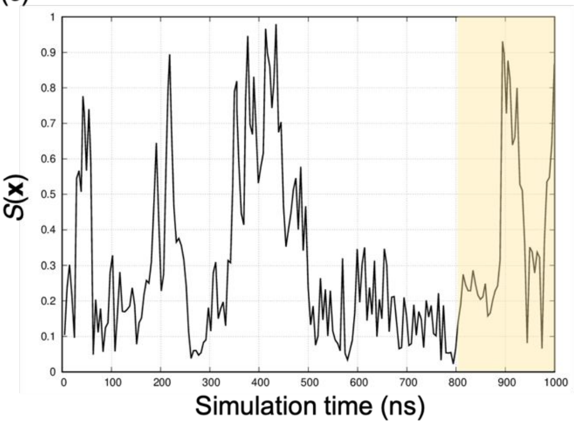
S(x)
Simulation time (ns)

(e)

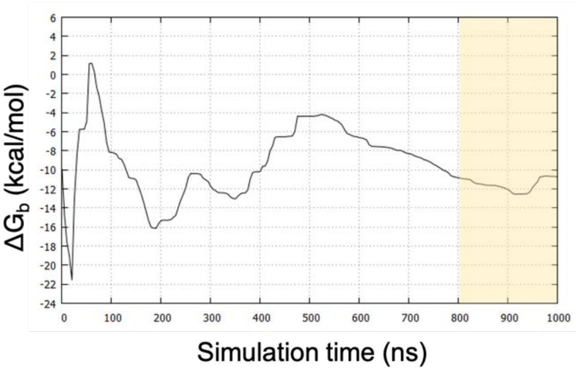
ΔG_b (kcal/mol)
Simulation time (ns)

6

(c)

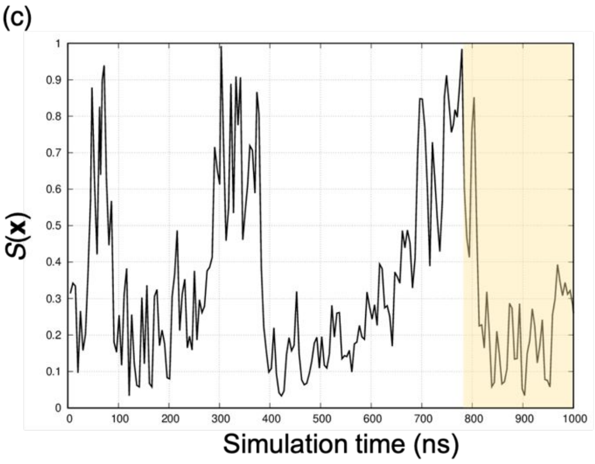
S(x)
Simulation time (ns)

(e)

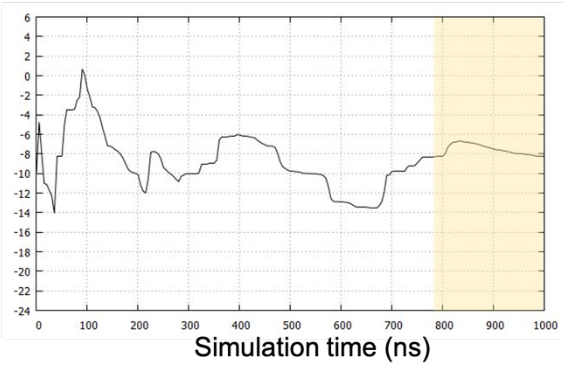
ΔG_b (kcal/mol)
Simulation time (ns)

7

(c)

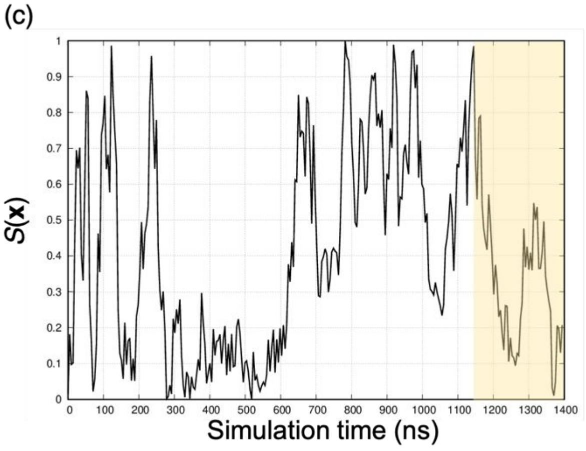
S(x)
Simulation time (ns)

(e)

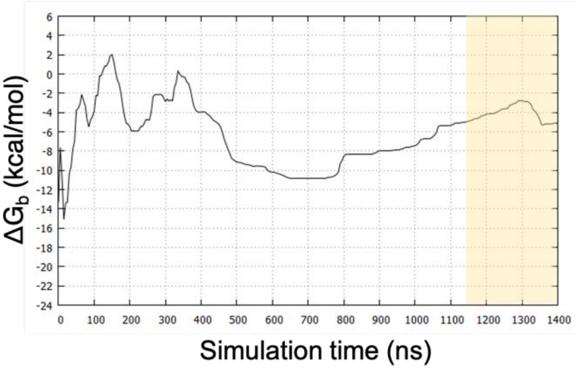
ΔG_b (kcal/mol)
Simulation time (ns)

8

(c)

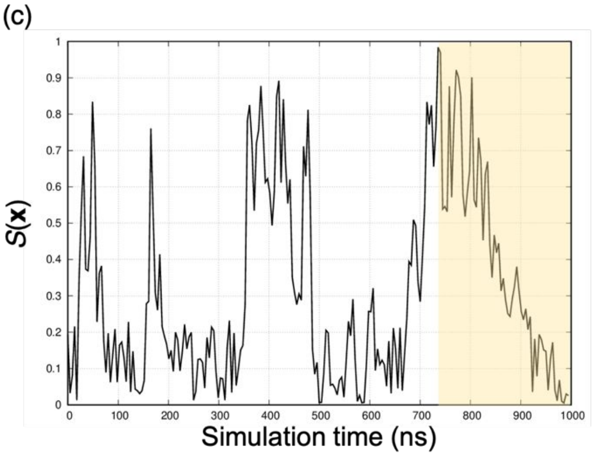
S(x)
Simulation time (ns)

(e)

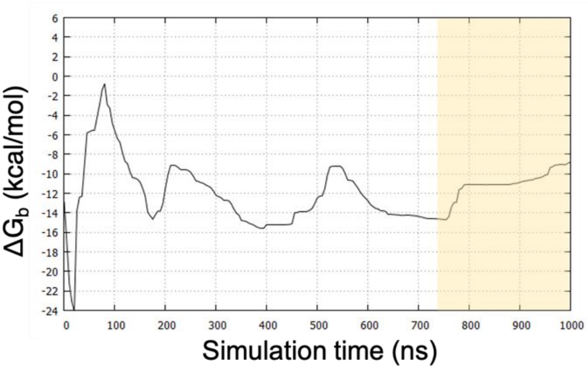
ΔG_b (kcal/mol)
Simulation time (ns)

Algorithm 1 Equidistant waypoints algorithm pseudo-code

Input

θ_0 initial path

t distance threshold between consecutive frames

Output

θ_n final path

```
Function equidistant_waypoints_algorithm(θ0,t)
TH = 0.001 (Angstroms)
α = θ0[0]
θn = []

for a in range(0,|θ0| −2):
        β = θ0[a + 1]
        γ = θ0[a + 2]
        σ = RMSD(α,β)

        while σ < t:
                a = a+1
                β = θ0[a + 1]
                γ = θ0[a + 2]
                σ = RMSD(α,β)
        g = σ/t

        while g ≥ 1:
                X = SMD(α,β,γ,t)
                r,φ = RMSD_nearest(α,X, t)

                if |r − t| < TH:
                    θn.append(φ)
                    α = φ
                    g = g − 1
                else:
                    while |r - t| > TH:
```

$X_t = \text{continue\_SMD}(\alpha,\beta,\gamma,t)$

$r,\varphi = \text{RMSD\_nearest}(\alpha,X_t,\beta)$

$\theta_n.append(\varphi)$

$\alpha = \varphi$

$g = g - 1$

SMD = steered MD simulation to drag β to a RMSD distance of about t from α, while minimizing the perturbation of the RMSD distance from γ.

Continue_SMD = extend the previous SMD simulation.

RMSD_nearest = from the SMD trajectory (X) returns the frame (φ) and the RMSD(r) closer to t from the target frame α.

References

1. Decherchi, S.; Bottegoni, G.; Spitaleri, A.; Rocchia, W.; Cavalli, A. BiKi Life Sciences: A New Suite for Molecular Dynamics and Related Methods in Drug Discovery. *J. Chem. Inf. Model.* **2018**, *58*, 219-224.

2. Decherchi, S.; Rocchia, W. A general and robust ray-casting-based algorithm for triangulating surfaces at the nanoscale. *PLoS ONE* **2013**, *8*, e59744-e59744.
